# Supplementary material for: Mutant p53 induces Golgi tubulo-vesiculation driving a prometastatic secretome
Source: Nat Commun. 2020 Aug 7;11:3945. doi: 10.1038/s41467-020-17596-5 (PMC7414119; doi:10.1038/s41467-020-17596-5)

SUPPLEMENTARY INFORMATION

Mutant p53 induces Golgi tubulo-vesiculation driving a prometastatic secretome

Capaci V., et al.

SUPPLEMENTARY FIGURES

Supplementary FIGURE1

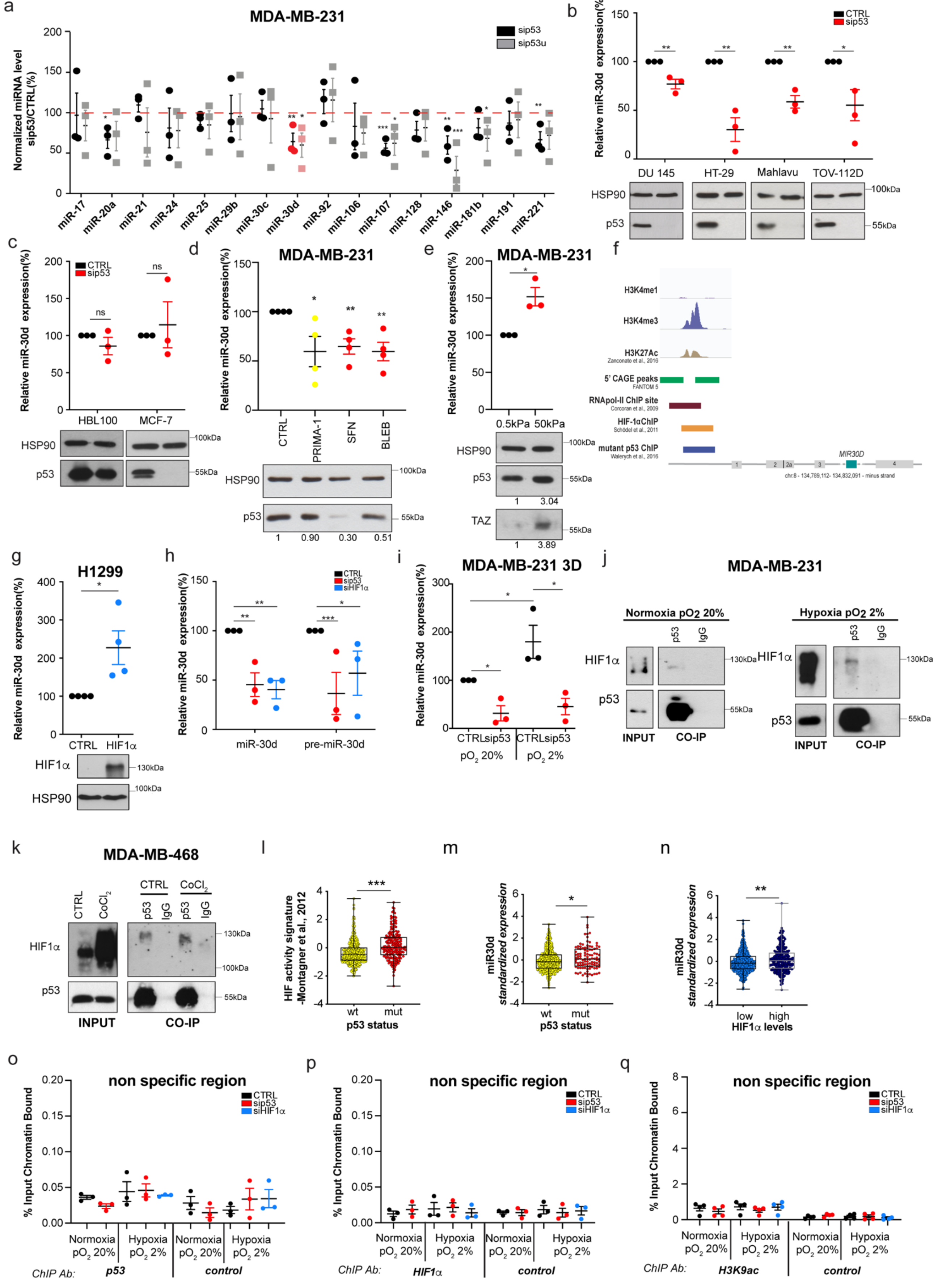

Supplementary Figure1. Mutant p53 induces miR-30d expression through HIF1α

a) mut-p53 was silenced in MDA-MB-231 cells with siRNAs targeting coding (sip53) or 3'UTR (sip53u) sequence. Expression of indicated miRNAs was measured by RT-qPCR normalized to U6B RNA.

b) c) RT-qPCR analysis of miR-30d expression in the indicated cell lines was performed as in **a**.

d) Analysis of miR-30d in MDA-MB-231 cells upon DMSO (CTRL), 10 μM PRIMA-1, 20 μM Sulforaphane (SFN), or 50 mM Blebbistatin (Bleb) treatment. Mut-p53 expression was normalized to Hsp90 quantified by densitometry.

e) Analysis of miR-30d in MDA-MB-231 cells on soft (0.5 kPa) or stiff (50 kPa) fibronectin-coated hydrogels for 7 days. Quantification of mut-p53 and TAZ was performed as in **d**.

f) Scheme of *MIR30D* locus, indicating promoter marks, putative TSS, and regions bound by RNApol II, HIF1α and mut-p53.

g) miR-30d expression analysis in H1299 cells upon HIF1α overexpression.

h) miR-30d and pre-miR-30d expression analyzed upon silencing mut-p53 or HIF1α.

i) miR-30d expression analyzed upon mut-p53 silencing in MDA-MB-231 cells grown in 3D Matrigel for 8 days, under normoxia or hypoxia (pO_2 2%).

j) Co-immunoprecipitation of mut-p53 and HIF1α in MDA-MB-231 cells cultured under normoxic or hypoxic (pO2 2%) conditions for 16h.

k) Co-immunoprecipitation of mut-p53 and HIF1α in MDA-MB-468 cells cultured in normoxic conditions or hypoxia-mimetic treatment (150μM $CoCl_2$) for 16h.

l) Average values of HIF1α activity signature[1] in breast cancer metadataset (see methods for details) samples stratified for p53 status.

m) Average expression of miR-30d in 781 breast cancer patients stratified for p53 status, obtained from the Molecular Taxonomy of Breast Cancer International Consortium, METABRIC[2].

n) Average expression of miR-30d in 1078 breast cancer patients stratified for HIF1α expression, obtained from The Cancer Genome Atlas (TCGA) breast cancer dataset.

o), p), q) Binding of p53, HIF1α and enrichment of Acetyl-Histone H3 (Lys9) to non-specific chromatin region from Fig.1 **g), h), i)**), calculated as fraction of input chromatin bound.

Graphs represent individual data points, mean +/- SEM of three independent experiments. Blots are representative of n=3 biological replicates. P value (* p< 0.05, ** p< 0.01, *** p < 0.001) by two-tailed unpaired Student's t-test. Source data provided as Source Data file.

Supplementary FIGURE2

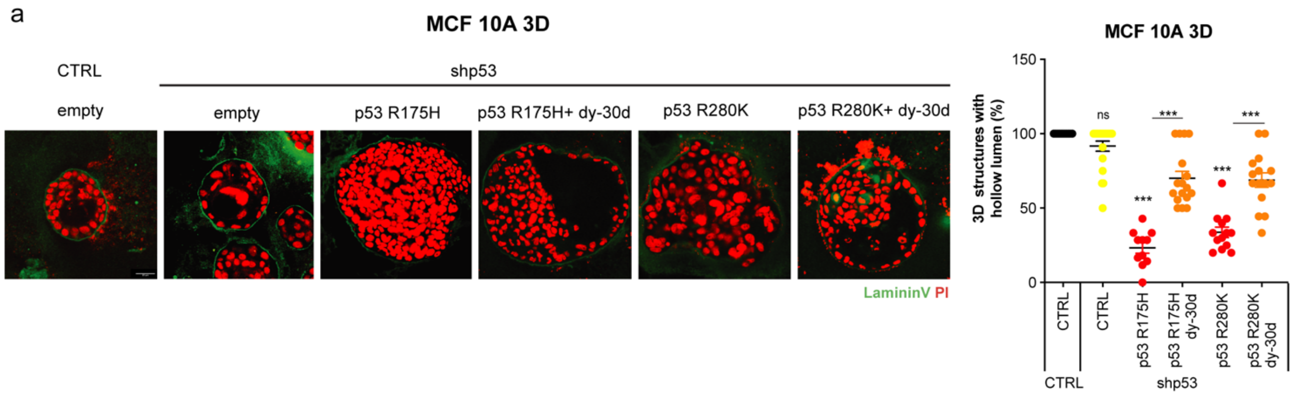

Supplementary Figure 2. miR-30d mediates mut-p53 dependent tumor phenotypes

a) Immunofluorescence analysis of Laminin V in MCF10A cells grown in three-dimensional Matrigel culture for 14 days. Cells were stably silenced for endogenous p53 (shp53) and transduced with constructs overexpressing either mut-p53 R175H or R280K forms, and either with control or miR-30d decoy construct as indicated. Scale bar, 20 μM. 3D structures were analyzed by optical microscopy and spherical cell clusters exhibiting inside lumen were categorized as "hollow lumen". The graph shows the number of structures with hollow lumen for field, mean +/- SEM of three independent experiments. n>65 structures were measured for each condition. (P value: ***p< 0.001 by two-tailed unpaired Student's t-test). Source data are provided as Source Data file.

Supplementary FIGURE 3

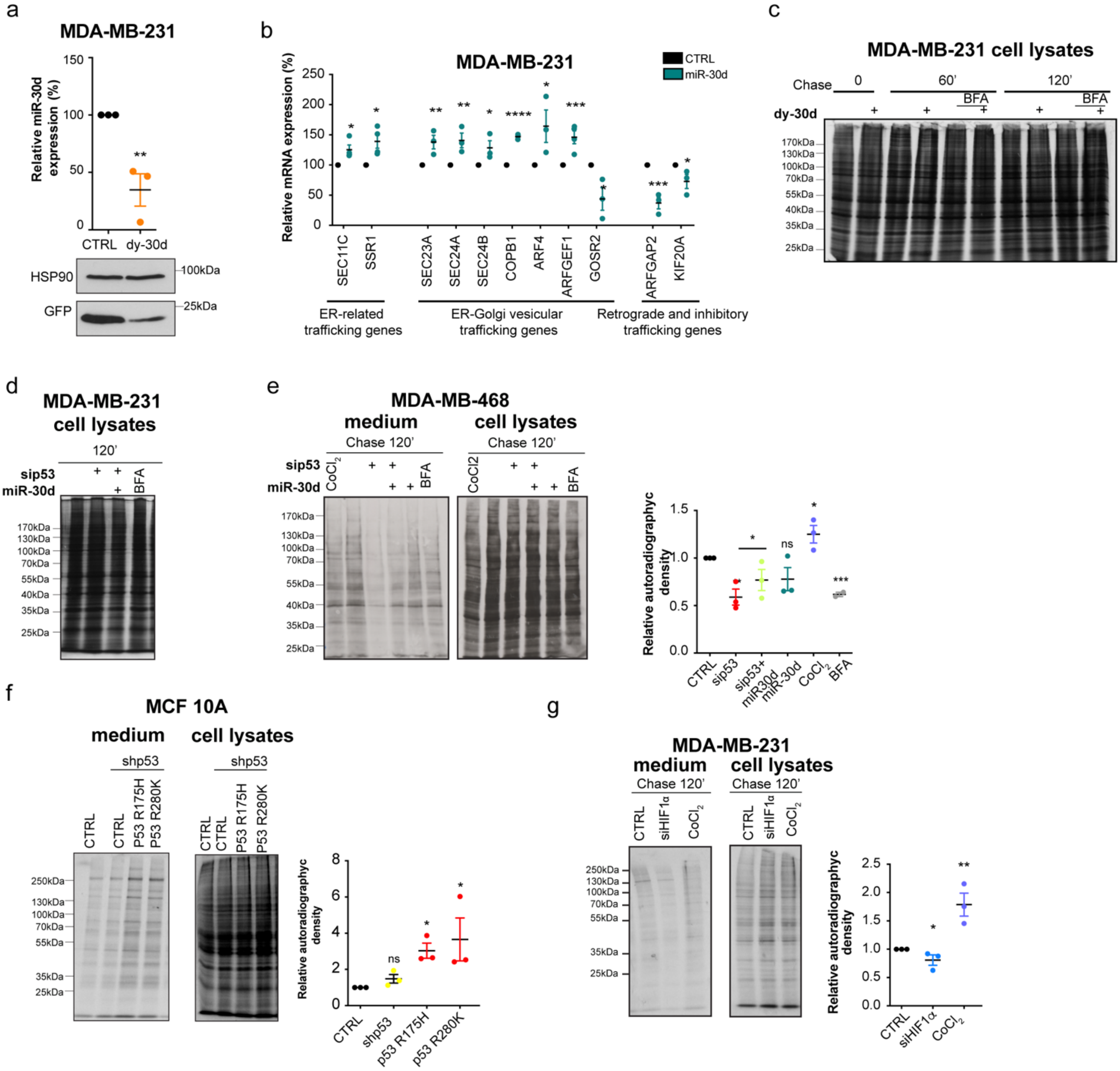

Supplementary Figure 3. mut-p53/HIF1α/miR-30d axis regulates protein secretion

a) Top: expression of miR-30d was evaluated by RT-qPCR in MDA-MB-231 cells stably transduced with Tween-GFP-3'UTR-miR-30d decoy lentiviral vector (dy-30d) or Tween-GFP empty vector. Bottom: western blot analysis of GFP expression using HSP90 as loading control (n=3, mean +/- SEM).

b) Expression of the indicated genes was evaluated in MDA-MB-231 cells transfected with either miR-30d mimic or control. The indicated mRNAs were analyzed by RT-qPCR normalized to H3 expression (n=3, mean +/- SEM).

c) Analysis of intracellular proteins relative to the experiment in Fig **2c.** MDA-MB-231 cells were transduced with miR-30d decoy vector and metabolically labeled with [^{35}S]-methionine/cysteine aminoacids. Labeled proteins were resolved by SDS-PAGE and detected by autoradiography. Treatment with 2.5 μM brefeldin A (BFA; an inhibitor of protein transport from the ER to the GA) was

used as control.

d) Analysis of intracellular proteins relative to the experiment in Fig **2d.** MDA-MB-231 cells were transfected with mut-p53 siRNA, miR-30d mimic, or a combination of them. Cells were treated and analyzed as in **c**.

e) Analysis of total secreted and intracellular proteins was performed after metabolic labeling as in **c,** using conditioned medium and cell lysate of MDA-MB-468 cells treated with $CoCl_2$ or transfected with mut-p53 siRNA, miR-30d mimic, or a combination of them as indicated. Right: graph showing ratio of secreted proteins (medium) to intracellular proteins (cell lysate) calculated by densitometry of the relative autoradiographs.

f) Analysis of total secreted and intracellular proteins was performed as in **e** with MCF10A cells in which endogenous wt-p53 was stably silenced (shp53) and replaced by overexpression of shRNA-resistant missense p53 mutants R175H and R280K.

g) Analysis of total secreted and intracellular proteins was performed as in **d** with MDA-MB-231 cells treated with $CoCl_2$ for 16h or transfected with HIF1α siRNA.

Graphs represent the individual data points, the mean +/- SEM of three independent experiments. Blots and autoradiographs shown are representative of n=3 biological replicates. P value: * $p< 0.05$, ** $p< 0.01$, *** $p < 0.001$ by two-tailed unpaired Student's t-test. Source data are provided as a Source Data file.

Supplementary FIGURE 4

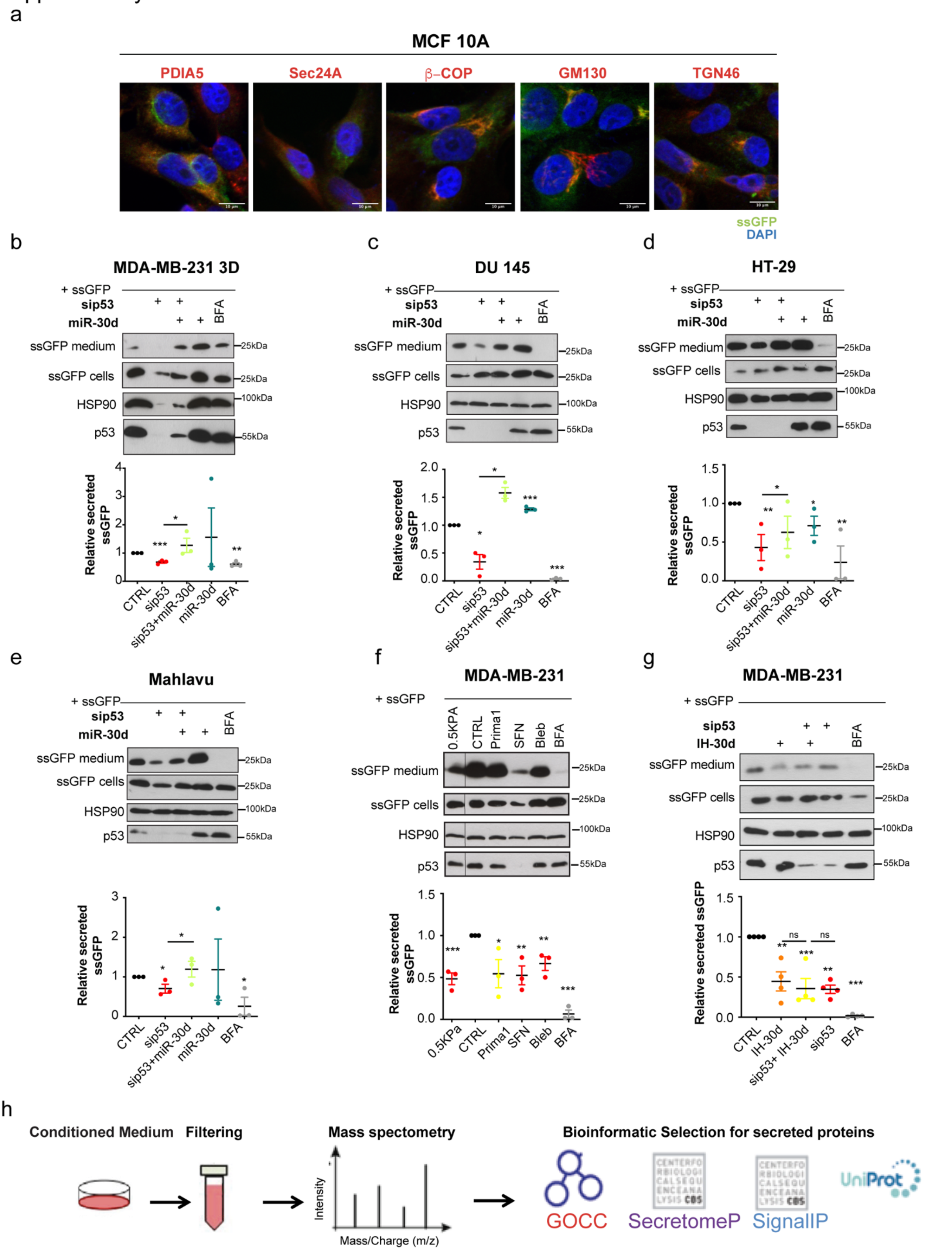

Supplementary Figure 4. mut-p53 and miR-30d regulate protein secretion

a) Immunofluorescence analysis of ssGFP and PDIA5 (ER), SEC24 (COPII), βCOP (COPI), GM130

(*cis*-Golgi), α-tubulin (microtubules) in MCF10A cells. Nuclei were stained with DAPI**.** Scale bar, 10 µm.

b) Intracellular and extracellular (medium) levels of ssGFP protein were analyzed in MDA-MB-231 cells grown in three-dimensional Matrigel culture for 8 days after silencing mut-p53, overexpression of a miR-30d mimic, or their combination. 48 h upon silencing, fresh medium was added to the cells and collected after 2h for analysis. Treatment with BFA 0.25 μM for 24h was performed as a control. Bottom: graph showing for each data point the ratio of secreted (medium) to intracellular (cells) ssGFP levels calculated by densitometry of the autoradiographs (average of 3 experiments).

c-d-e) Intracellular and extracellular (medium) levels of ssGFP protein were analyzed as in **b** in DU 145, HT-29 and Mahlavu cells grown in 2D culture.

f) Intracellular and extracellular (medium) levels of ssGFP protein were analyzed as in **b** in MDA-MB-231 cells grown on soft (0.5 kPa) or stiff (50 kPa) fibronectin-coated hydrogels for 7 days, or treated for 24h with DMSO (CTRL), 10 µM PRIMA-1, 20 µM Sulforaphane (SFN), or 50 mM Blebbistatin (Bleb).

g) Intracellular and extracellular (medium) levels of ssGFP protein were analyzed as in **b** in MDA-MB-231 cells upon transfecting mut-p53 siRNA, miR-30d inhibitor (IH-30d), or their combination as indicated.

h) Schematic representation of the analysis of mutp53-dependent secretome. MDA-MB-231 cells were transfected with mut-p53 siRNA, alone or in combination with miR-30d mimic; CM was collected after 72h, filtered by 0.20 μm syringe filters to remove cell debris, and subjected to LC-MS/MS. All positive hits were categorized using the indicated bioinformatic tools to select proteins that are released either via the classical ER−Golgi secretory pathway or via alternative non-canonical pathways, as previously described by [3] using GOCC[4,5]; SecretomeP[6] ; SignalP[7]; UniProt[8] tools.

Graphs represent individual data points, mean +/- SEM of three independent experiments. Blots and micrographs are representative of n=3 biological replicates. P value: * $p< 0.05$, ** $p< 0.01$, *** $p < 0.001$ by two-tailed unpaired Student's t-test. Source data are provided as Source Data file.

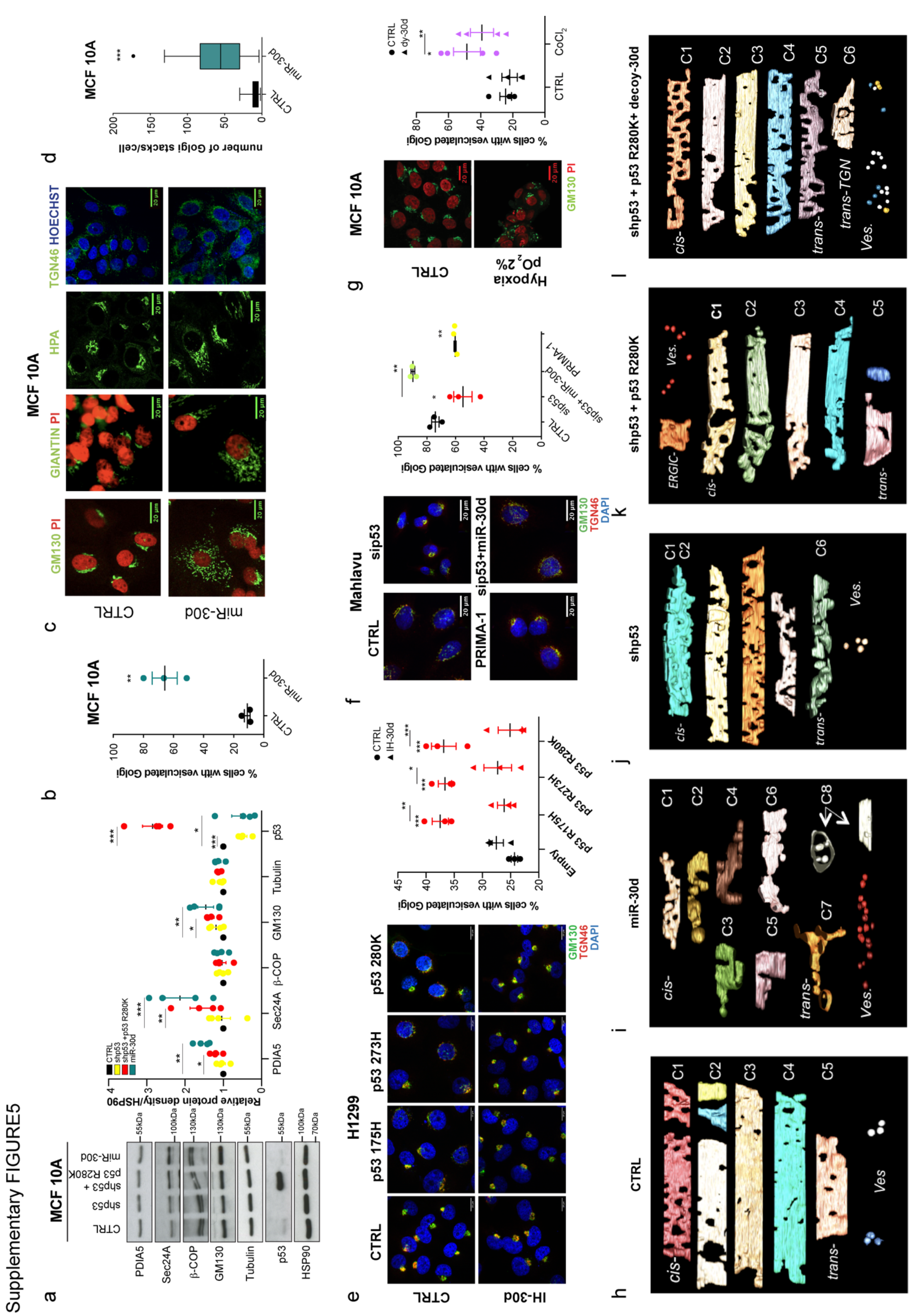

Supplementary Figure 5. mut-p53/miR-30d modify the cell secretory machinery.

a) Western blot analysis of PDIA5, SEC24, βCOP, GM130 and α-tubulin in MCF10A cells upon stable silencing of wt-p53 (shp53) alone or combined with overexpression of mut-p53 R280K or miR-30d mimic. HSP90 was used as loading control. The blot is representative of n=3 biological repeats. Right: expression of reported proteins relative to HSP90 quantified by densitometry.
b) Graph showing the percentage of MCF10A cells with vesiculated Golgi upon overexpression of miR-30d as compared to control, calculated upon immunofluorescence with GM130 staining.
c) Immunofluorescence analysis of Golgi apparatus morphology in MCF10A cells transfected with control or miR-30d mimic. Golgi was stained with antibodies specific for Golgi markers GM130, giantin, TGN46, or with *Helix pomatia* agglutinin-Alexafluor 488 (HPA) conjugated (Scale bar, 20 μm).
d) Quantification of Golgi elements in MCF10A cells, transfected with control or miR-30d mimic, as calculated by Volocity in confocal Z-stack images of cells stained with GM130 antibody (n=50 cells were measured for each condition). Box shows median and whiskers of 3 independent experiments calculated with Tukey method. (Two-tailed unpaired t test *p< 0.05).
e) Immunofluorescence analysis of Golgi apparatus stained with GM130 antibody in H1299 cells overexpressing mut-p53 R175H, R273H or R280K, upon transfection of miR-30d inhibitor (IH-30d). Scale bar, 20 μm. Right: graph showing the percentage of cells with vesiculated Golgi upon different treatments.
f) Analysis of Golgi apparatus was performed as in **e)** in Mahlavu cells upon silencing mut-p53, overexpression of miR-30d mimic, their combination or treatment for 24h with 10 μM PRIMA-1. Scale bar, 20 μm. Right: graph showing the percentage of cells with vesiculated Golgi.
g) Left: immunofluorescence analysis of Golgi apparatus was performed as in **e)** in MCF10A cells transduced with control or miR-30d decoy, and treated with $CoCl_2$ for 16h (Scale bar, 20 μm). Right: percentage of cells with vesiculated Golgi.
h-k) Three-dimensional models of single cisternae forming the stack shown in Fig.**3c**.
Graphs represent individual data points, mean +/- SEM of three independent experiments. P value: * p< 0.05, ** p< 0.01, *** p < 0.001 by two-tailed unpaired Student's t-test. Source data are provided as Source Data file.

Supplementary FIGURE 6

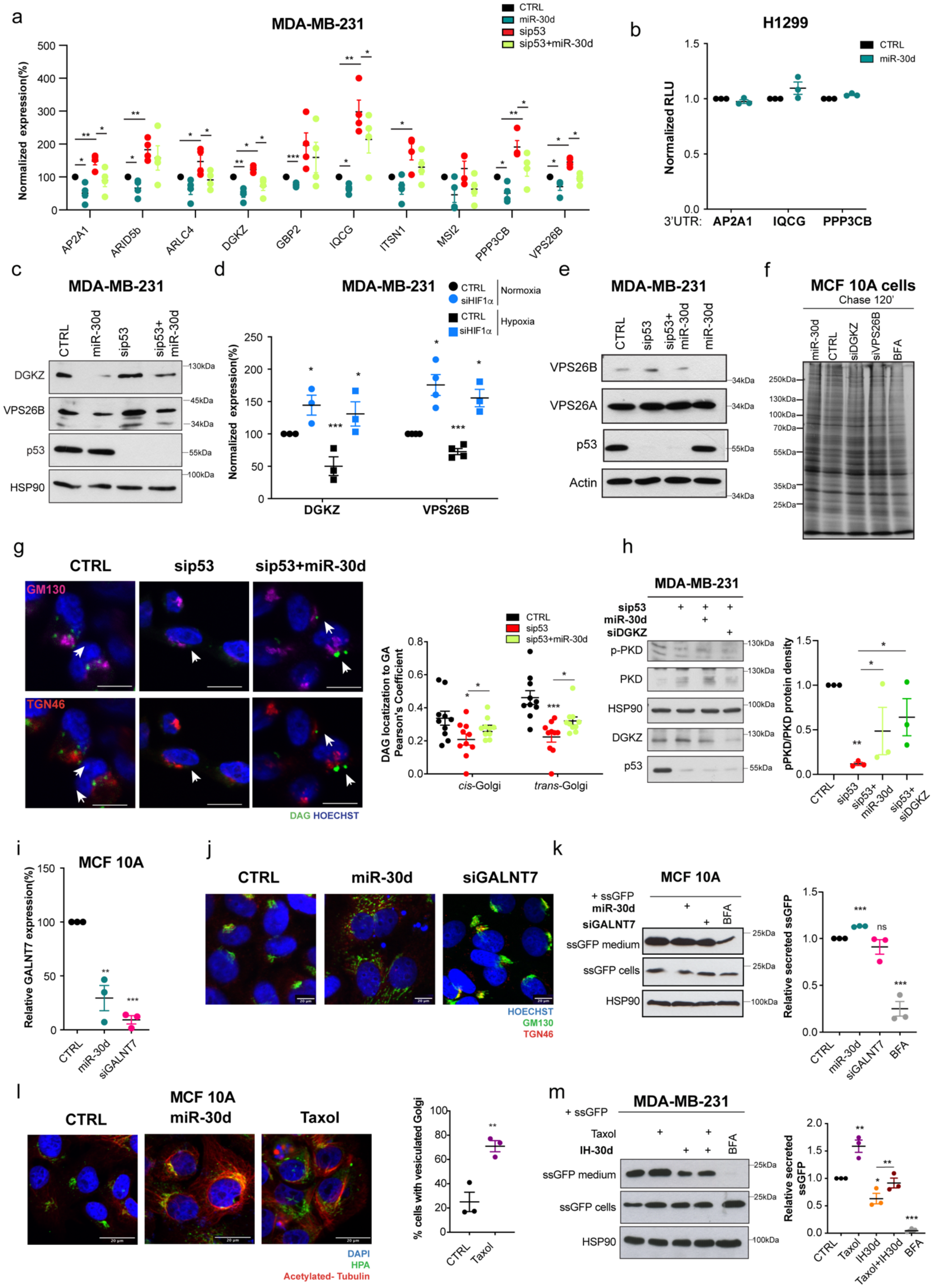

Supplementary Figure 6. miR-30d alters the secretory pathway via DGKZ and VPS26B

a) RT-qPCR analysis of indicated mRNAs in MDA-MB-231 cells transfected with miR-30d mimic, mut-p53 siRNA or combination.
b) Indicated 3'UTR-luciferase reporter assays were performed in H1299 cells transfected with control (CTRL) or miR-30d mimic.
c) WB analysis of DGKZ, VPS26B and p53 in MDA-MB-231 cells transfected with mut-p53 siRNA and/or miR-30d mimic.
d) Expression of DGKZ and VPS26B mRNA upon silencing HIF1α in MDA-MB-231 cells in normoxia or hypoxia (pO_2 2%), evaluated as in **a**.
e) Analysis of VPS26A, VPS26B, and p53 protein levels in MDA-MB-231 cells treated as in **c**.
f) Analysis of intracellular proteins of the experiment in Fig **4f**.
g) Co-localization of DAG with Golgi markers GM130 and TGN46 in MDA-MB-231 cells transfected with mut-p53 siRNA and/or miR-30d mimic, was analyzed with a construct encoding C1A-PKCγ DAG-binding domain fused to eGFP. Right: Pearson's correlation coefficient (n=10 cells/condition).
h) Analysis of PKD and pPKD in MDA-MB-231 cells transfected with mut-p53 siRNA, miR-30d mimic, and DGKZ siRNA or indicated combinations.
i) Expression of GALNT7 mRNA in MCF10A cells overexpressing miR-30d mimic or silencing GALNT7 was evaluated as in **a**.
j) Immunofluorescence analysis of Golgi morphology (stained for GM130) in MCF10A cells as in **h**.
k) Intracellular and secreted ssGFP levels were analyzed in MCF10A cells stably transduced with ssGFP construct upon overexpressing miR-30d mimic or silencing GALNT7. 48h upon silencing, fresh medium was added to cells and collected after 2h for analysis. Treatment with BFA 2.5 μM for 2h was included as control. Right: Relative secreted ssGFP normalized to control.
l) Immunofluorescence analysis of Golgi morphology and acetylated-tubulin in MCF10A cells upon miR-30d overexpression or treatment with Taxol (1μM for 2h). The graph shows percentages of cells with vesiculated GA.
m) Intracellular and secreted ssGFP levels analyzed as in **k** in MDA-MB-231 cells upon miR-30d inhibition and/or Taxol treatment (1μM for 2h).
Graphs represent the individual data points and the mean +/- SEM of three independent experiments (*p< 0.05, **p< 0.01, *** p < 0.001, ns=not significant by unpaired two-tailed Student's t-test).
Blots and autoradiographs are representative of n=3 biological repeats. Source data are provided as Source Data file.

Supplementary FIGURE 7

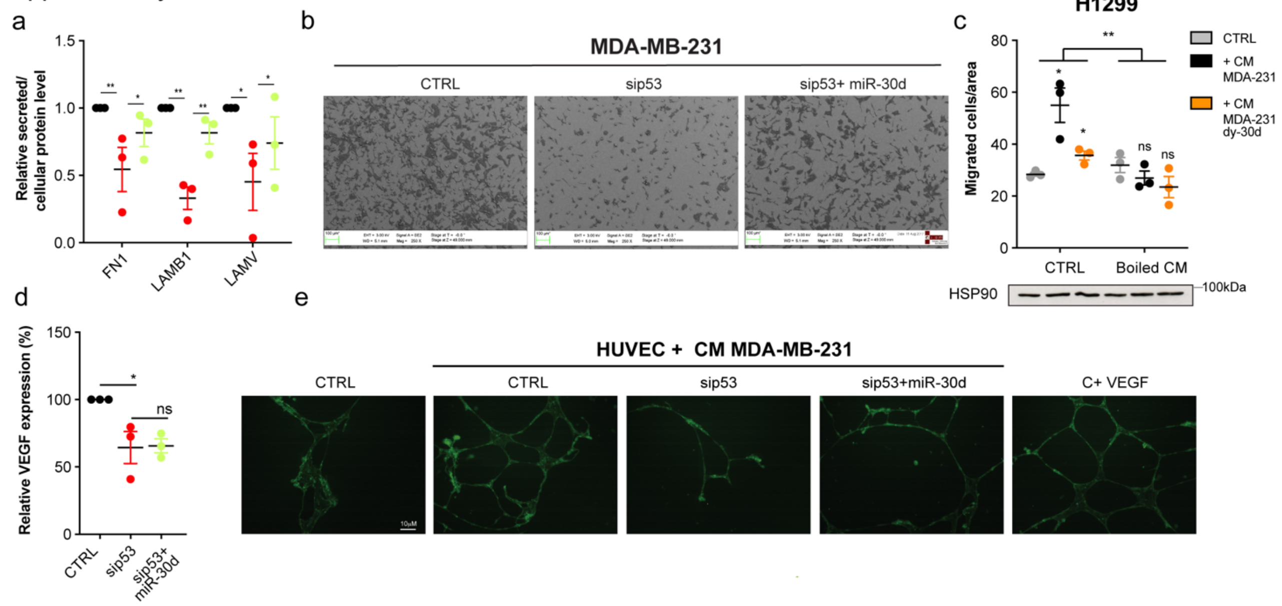

Supplementary Figure 7. The mut-p53/miR-30d secretome induces a supportive TME

a) Graph showing the ratio of secreted versus intracellular FN1, Lamb1 and LamV from Fig. 5b quantified by densitometry in MDA-MB-231 cells upon transfection with mut-p53 siRNA (sip53) alone or combined with miR-30d mimic.

b) Scanning electron microscopy analysis of MDA-MB-231 cells and of their deposited ECM, upon transfection with mut-p53 siRNA, miR-30d mimic or their combination. Fibrillar collagens were stained with PicroSirius, and cells counterstained with hematoxylin.

c) Cell migration analysis of H1299 cells, previously treated for 48h with either unheated or heat-inactivated (boiled) conditioned DMEM medium, obtained from MDA-MB-231 cells transduced either with control vector (CTRL) or miR-30d decoy (dy-30d). Cell migration was assayed by transwelling (Boyden chamber) for 16 hours. Conditioned DMEM medium obtained from untreated H1299 cells was used as control (grey bars). Bottom: inhibition of dy-30d and relative amounts of H1299 cells in different experimental points were controlled by Western blot.

d) Expression of VEGF in MDA-MB-231 cells upon silencing mut-p53, overexpressing miR-30d mimic or their combination was evaluated by RT-qPCR normalized to H3 expression, and compared to control-transfected cells.

e) Representative images of endothelial closed loops formed by HUVEC cells 7hr after plating on Matrigel in presence of their own medium (CTRL) or of medium conditioned (CM) by MDA-MB-231 cells transfected with mut-p53 siRNA alone or combined with miR-30d mimic. Cells were stained with FITC-phalloidin. VEGF treatment was used as a positive control.

Graphs represent the individual data points and the mean +/- SEM of three independent experiments (*$p< 0.05$, **$p< 0.01$, *** $p < 0.001$, ns not significant by unpaired two-tailed Student's t-test).

Blots are representative of n=3 biological repeats. Source data are provided as a Source Data file.

Supplementary FIGURE 8

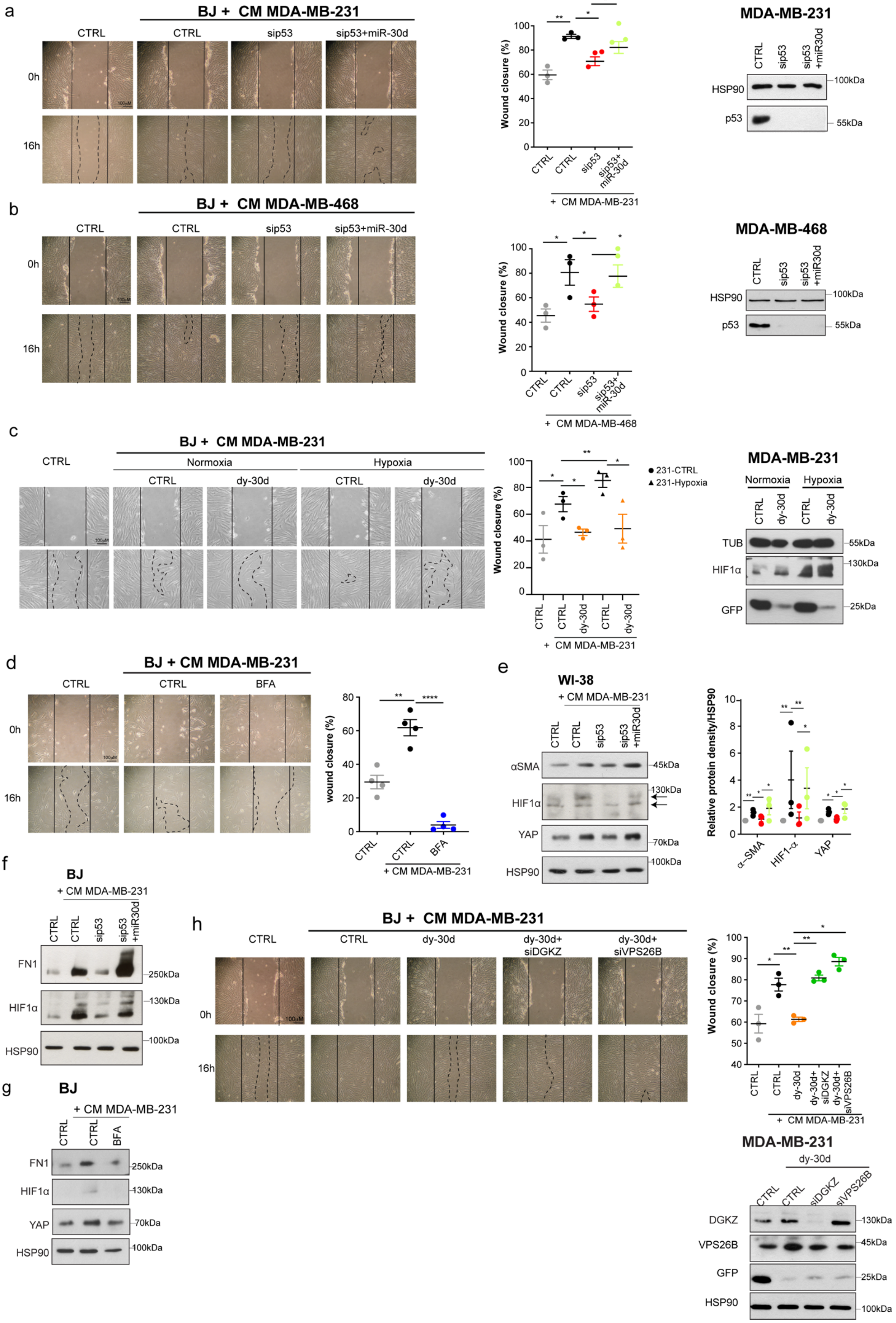

Supplementary Figure 8. The mut-p53/miR-30d secretome activates CAFs

a) Left: Representative light microscopy images of wound healing assays performed with BJ-EHT-ER-RAS (BJ) human fibroblasts treated with their own medium (CTRL) or with CM by MDA-MB-231 cells transfected with mut-p53 siRNA, alone or combined with miR-30d mimic. The graph shows the percentage of wound closure. Right: p53 levels in MDA-MB-231 cells used to produce CM.
b) Wound healing assays were performed as in **a**, using CM by MDA-MB-468 cells. Right: p53 levels in MDA-MB-468 cells used to produce CM.
c) Wound healing assays performed as in **a** with BJ fibroblasts treated with their own medium (CTRL) or with CM by MDA-MB-231 cells cultured either in normoxic or hypoxic conditions (pO_2 2% for 48h) combined with miR-30d inhibition by decoy construct. Right: HIF1α and GFP expression in MDA-MB-231 cells used to produce CM.
d) Wound healing assays performed as in **a** with BJ fibroblasts treated with their own medium (CTRL) or CM by MDA-MB-231 cells either treated or not with BFA 0,25 μl for 72h.
e) Analysis of CAF activation markers in BJ fibroblasts treated with their own medium or with CM by MDA-MB-231 cells transfected as in **a**.
f) Analysis of CAF activation markers in WI-38 fibroblasts treated with their own medium or with CM by MDA-MB-231 cells (CM) from Fig. 5**i-j**. The graph shows the expression of α-SMA, HIF1α and YAP relative to HSP90 quantified by densitometry. Arrows indicate HIF1α protein bands.
g) Analysis of CAF activation markers in BJ fibroblasts treated with their own medium or with CM by MDA-MB-231 cells as in **i**.
h) Wound healing assays performed with BJ fibroblasts in presence of their own medium or CM by MDA-MB-231-dy-30d cells, and transfected either with DGKZ or VPS26B siRNAs. Right: analysis of DGKZ, VPS26B and GFP levels in MDA-MB-231 cells used to produce CM**.** HSP90 was used as loading control.
Graphs represent the individual data points and the mean +/- SEM of three independent experiments (*p< 0.05, **p< 0.01, *** p < 0.001, by unpaired two-tailed Student's t-test).
Blots are representative of n=3 biological repeats. Source data are provided as a Source Data file.

Supplementary FIGURE9

a

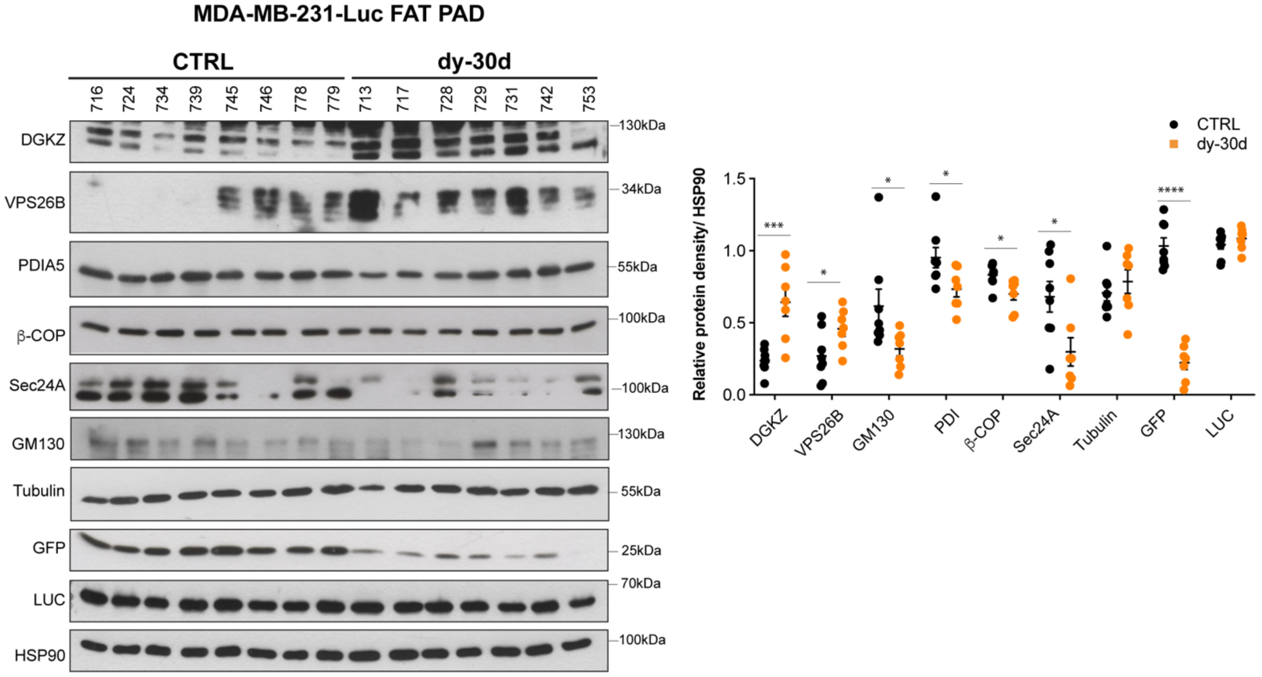

b

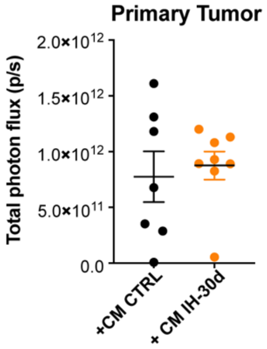

Supplementary Figure 9. miR-30d enhances tumorigenesis and metastasis *in vivo*

a) Left: Western blot analysis of the indicated proteins in primary tumors from mice in Figure **6a-b.** Mice were engrafted with MDA-MB-231-Luc cells transduced with either control (CTRL) or miR-30d decoy (dy-30d) construct. HSP90 was used as loading control. Right: graph shows the quantification of autoradiographic bands measured by densitometry, normalized to HSP90.

b) Whole body *in vivo* luciferase quantification of primary tumors before surgical resection after 10 days of orthotopic xenograft of MDA-MB-231–Luc cells in SCID mice from Fig. **6e (**CTRL n=8, dy-30d n=9).

Graphs represent the individual data points and the mean +/- SEM of three independent experiments (*p< 0.05, **p< 0.01, *** p < 0.001, by unpaired two-tailed Student’s t-test).

Blots are representative of n=3 biological repeats. Source data are provided as a Source Data file.

SUPPLEMENTARY TABLES

Supplementary Table1. TP53 status of breast cancer patients used to determine correlation between the p53 status, miR-30d levels and secretory pathway (Fig. 7e-k).

	p53 hot spot mutation:										p53 IHC:	
	c.488A>G p.Y163C	c.517G>T p.V173L	c.524G>A p.R175H	c.535C>T p.H179Y	c.659A>G p.Y220C	c.711G>T p.M273I	c.742C>T p.R248W	c.743G>A p.R248Q	c.818G>A p.R273H	c.818G>T p.R273L	low	high
Patient 1											x	
Patient 2											x	
Patient 3											x	
Patient 4											x	
Patient 5											x	
Patient 6											x	
Patient 7				x								x
Patient 8									x			x
Patient 9			x									x
Patient 10								x				x
Patient 11								x				x
Patient 12							x					x

Supplementary Table 2. List of the primers used in the present study.

**qPCR primers miRNAs**		
**Official Symbol**	**Accession Number**	**miScript Primer Assay Catalog Number (Qiagen)**
SNORD25	NR_002565.1	MS00014007
RNU6B	NR_004394.1	MS00014000
hsa-miR-17-5p	MI000007	MS00008820
hsa-miR-20a	MI0000076	MS00003199
hsa-miR-21	MI0000077	MS00009079
hsa-miR-24	MI0000080	MS00006552
hsa-miR-25	MI0000082	MS00003227
hsa-miR-29b	MI0000105	MS00006566
hsa-miR-30c	MI0000736	MS00009366
hsa-miR-30d	MI0000255	MS00009387
hsa-miR-92	MI0000093	MS00006594
hsa-miR-106a	MI0000113	MS00003395
hsa-miR-107	MI0000114	MS00003409
hsa-miR-128b	MI0000727	MS00008582
hsa-miR-146a	MI0000477	MS00003535
hsa-miR-181b	MI0000270	MS00006699
hsa-miR-191	MI0000465	MS00003682
hsa-miR-221	MI0000298	MS00003857
hsa-pre-miR-30d	MI0000255	MP00001848
**qPCR primers mRNAs**		
**Gene Name:**	**Primer sequence:**	**Direction:**
ARF4	GGCCTCTCTGGAAGCATTACT	FW
ARF4	AGCAGCACTGCATCTCTCAAT	REV
ARFGAP2	TGGCTAGGCATGGCACTG	FW
ARFGAP2	GGCTTCTTCTTGCCAATGATGG	REV
ARFGEF1	AGCGCGATCTGGTACAAACT	FW
ARFGEF1	TCACTTACAGGAGATGGAGGT	REV
COPB1	TGGGCCGAATTTGAATGGGA	FW
COPB1	CGAGCATAAAGGTTGGCTGC	REV
GOSR2C	CACCACTAACGACTCTGACACCAC	FW
GOSR2C	ATCCCAAACTCAAGGGCTCTGTC	REV
KIF-20A	CTCCCTAGAGGACAAGCAGC	FW
KIF-20A	TACGGACACAACCCTGATCT	REV
SEC11C	GCGTCCGGCTTGGATATCTT	FW
SEC11C	GTCTCCTCTGTGAAAGGCCG	REV
SEC23A	AGTCAACCCTTCACAGACTCAT	FW
SEC23A	AGCACTGGACACAGCAAGTTT	REV
SEC24A	CGGTCTTCAGGAAAGTCTCTCT	FW
SEC24A	ATGGCAATTTCCAGGCAGGTA	REV
SEC24B	GCTGTAGCGAACAACAACCC	FW
SEC24B	CACAGGCTCAACTGGGTGAT	REV
SSR1	TCACAGCTCTTCCTCTGAACAC	FW
SSR1	CCAAACCAAATGGTCGTCCG	REV
AP2A1	TCATCTCCGACATCCGGAAC	FW

AP2A1	TCCAAGGCTTTGTCTCCTTTGA	REV
ARID5B	CACCTTTGACCACCCGACTC	FW
ARID5B	ATTTCACCTTGGCAACGGCT	REV
ARL4C	GAAACGCAGGAAGTCCCTCA	FW
ARL4C	TTGGTTCGCTCTTTGTTCGC	REV
DGKZ	AGCAGTACTGTGTAGCCAGGAT	FW
DGKZ	CACGGAAGGACGGCTTACAG	REV
GBP2	CTGCACAGGGACAGTGAGAG	FW
GBP2	AGTCATCTCGCCTTGCTTCC	REV
IQCG	CGAACTCACTGAGCTGGAAGT	FW
IQCG	AGTCTTCCAGGCTGTCTTCTTC	REV
ITSN1	GGTCCACTGCAGAAAAAGGTC	FW
ITSN1	GGGTTCTCCAGTTTGGCTTTC	REV
MSI2	AGCAAGAGGATCAGGCTCCA	FW
MSI2	GCCGTTGCAATCAAAGGTCC	REV
PPP3CB	CTCTGTTCTCAGGGAGGAGAGT	FW
PPP3CB	TCAGCCTCAATAGCCTCAACTG	REV
VPS26B	TTGGGATTGAGGACTGTCTGC	FW
VPS26B	TTCTCATGGTACACGTTGGGG	REV
Actin	CGCCGCCAGCTCACCATG	FW
Actin	CACGATGGAGGGGAAGACGG	REV
H3	GAAGAAACCTCATCGTTACAGGCCTGGT	FW
H3	CTGCAAAGCACCAATAGCTGCACTCTGGAA	REV
pri-miR-30d	ACTACCACCCAGCAGTATGC	FW
pri-miR-30d	GTGAGGGGAACAGGAAGTGG	REV
**Cloning Primers**		
**Target**	**Sequence**	**Fw/Rev**
PPP3CB 3'UTR	CATCATGACGTCCCCACTACTTCCCAGG	FW
PPP3CB 3'UTR	CATCATCATATGTGCAATTATCACTAATATTTTTCTTATTGT	RV
ITSN1 3'UTR	CATCATGACGTCTCATATGTTGTCCATCCCC	FW
ITSN1 3'UTR	CATCATACCGGTTAACTTTTGTATGAAGAATTTAATTATCTATACAC	RV
ITSN1 3'UTR	CATCATGACGTCGCAGCGGGCTCA	FW
ITSN1 3'UTR	CATCATACCGGTGCTACCTACATGAACAATGATG	RV
DGKZ 3'UTR	CATCATGACGTCACGAGCGCCTTCC	FW
DGKZ 3'UTR	CATCATCATATGAAAGAAAAAAAAATCCACTTTACTGAG	RV
MSI2 3'UTR	CATCATACCGGTGCAGGTGCTTTCGTTG	FW
MSI2 3'UTR	CATCATGGCCGGCCGTCAGACAAACACATTGATTTCT	RV
AP2A1 3'UTR	CATCATGACGTCCCCTGGACTCTGCC	FW
AP2A1 3'UTR	CATCATCATATGTAGCGTCTCTCTGTTTATTCG	RV
ARID5B 3'UTR	CATCATGACGTCGCTCTGCCCAGCA	FW
ARID5B 3'UTR	CATCATACCGGTGAAACAGATAGTAACATTTTTTATTAAATATTACAG	RV

GBP2 3'UTR	CATCATGACGTCGTCCAAGGAGCAAAAGC	FW
GBP2 3'UTR	CATCATACCGGTTAAAGTCTTCCAACAGTTTATTAGAAAG	RV
IQCG 3'UTR	CATCATGACGTCCCAAGTTCCTTGTGTTCTG	FW
IQCG 3'UTR	CATCATCATATGATGGTTTACAGCTTTCGTTTTAT	RV
Annealing of the rat FSHbeta ss	AGCTTATGATGAAGTCGATCCAGCTTTGCATCCTACTCTGGTGCTTGAGAGCAGTCTGCTGCCAT	UP
Annealing of the rat FSHbeta ss	GATCCATGGCAGCAGACTGCTCTCAAGCACCAGAGTAGGATGCAAAGCTGGATCGACTTCATCAT	DW
eGFP	CATCATGGATCCGTGAGCAAGGGCGAG	FW
eGFP	CATCATGAATTCTACTTGTACAGCTCGTCCA	RV
Decoy-30d	5´-TCGAG CTT CCA GTC GGG GAT GTT TAC AAGAGAACTTAGAGAACTT CTT CCA GTC GGG GAT GTT TACA T-3´	UP
Decoy-30d	5´-CTAGA TGT AAA CAT CCC CGA CTG GAA GAAGTTCTCTAAGTTCTCT TGT AAA CAT CCC CGA CTG GAAG C-3´	DW
**ChIP Primers:**		
**Gene Name:**	**Primer sequence:**	**Direction:**
MIR30D ChIP Binding	TATCTTGACACTTGAAGGCCCC	FW
MIR30D ChIP Binding	GCCTTGGCTGCCTGTGATA	REV
AchR ChIP heterochromatin	CAACCAAAGCCCATGTCCTC	FW
AchR ChIP heterochromatin	AGGCACGCTACAGGGCTTC	REV

Supplementary Table 3. List of the antibodies used in the present study.

Target protein name:	Producer:	ID number:	WB Dilution:	IF Dilution:	IHC Dilution:
Actin	Sigma-Aldrich	A2066; RRID: AB_476693	1:5000		
CD31	Abcam	ab124432; RRID:AB_2802125			1:2000
βCOP	Abcam	ab2899; RRID: AB_2081300	1:1000	1:50	
COL6A2	Invitrogen	PA5-65222; RRID:AB_2662555			1:100
DGKZ	Sigma-Aldrich	HPA051336; RRID: AB_2681448	1:1000		
Fibronectin (N1N2)	GeneTex	GTX112794; RRID: AB_1950298	1:1000		
FREM2	Abcam	ab117612; RRID: AB_10933708			1:200
GAPDH (6C5)	Santa Cruz Biotechnology	sc-32233; RRID: AB_627679	1:5000	1:100	
Giantin	Abcam	ab80864; RRID: AB_10670397			
GFP	home-made rabbit polyclonal antibody	N/A	1:1000	1:100	
GM130	BD	610822; RRID: AB_10015242			
HIF-1α (D2U3T)	Cell Signaling Technology	#14179; RRID: AB_2622225	1:1000		
HIF-1α (H1alpha67)	Novus Biologicals	NB100-105; RRID: AB_10001154			
HSP90 alpha/beta (F-8)	Santa Cruz Biotechnology	sc13119; RRID: AB_675659	1:5000		
Ki67 (Sp6)	Abcam	ab16667; RRID: AB_302459			1:5000
Laminin 5 (γ2 chain)	Millipore	MAB19562; RRID: AB_94454	1:1000		
Laminin-b1	Abcam	ab69633; RRID: AB_1269284	1:1000		
Laminin beta-3 (CL3363)	Invitrogen	MA524655; RRID: AB_2637270			1:200
Luciferase	Thermo Fisher Scientific	pa1-46333; RRID: AB_1090406	1:10000		
p53 DO-1	Santa Cruz Biotechnology	sc-126; RRID: AB_628082	1:2000		
p53	home-made rabbit polyclonal antibody	N/A	1:2000		
p53 FL-393	Santa Cruz Biotechnology	sc-6243; RRID: AB_653753	1:2000	1:100	
p53 (DO-7)	Leica Biosystems	NCL-L-p53-DO7; RRID: AB_563936			1:50
PDIA5 (RL90)	Abcam	ab2792; RRID: AB_303304		1:50	
PKD/PKCμ (D4J1N)	Cell Signaling Technology	#90039; RRID: AB_2800149	1:1000		

Phospho-PKD Ser744/748	Cell Signaling Technology	#2054; RRID: AB_2172539		1:100	
SEC24A	Abcam	ab102660; RRID: AB_10711917	1:1000	1:50	
a-SMA	Abcam	ab5694; RRID: AB_2223021	1:1000	1:200	1:400
α Tubulin	Sigma-Aldrich	T5168; RRID: AB_477579	1:2000	1:100	
Acetylated-Tubulin	Sigma-Aldrich	T6793; RRID: AB_477585		1:100	
TGN46	Abcam	ab50595; RRID: AB_2203289			
VEGFA	GeneTex	GTX102643; RRID: AB_11174248	1:1000		
VPS26A	Abcam	ab23892; RRID: AB_2215043	1:1000		
VPS26B	Sigma-Aldrich	SAB4500246; RRID: AB_10743760	1:1000		
WWTR1	Sigma-Aldrich	HPA007415; RRID: AB_1080602	1:1000		
YAP (H-125)	Santa Cruz Biotechnology	sc-15407; RRID: AB_2273277	1:1000		
Mouse normal IgG	Santa Cruz Biotechnology	sc-2025; RRID: AB_737182	-		
Rabbit normal IgG	Santa Cruz Biotechnology	sc-2027; RRID: AB_737197	-		
Mouse IgG-heavy and light chain cross-adsorbed antibody	Bethyl Laboratories Inc.	A90-516P; RRID: AB_10631212	1:10000		
Rabbit IgG-heavy and light chain cross-adsorbed antibody	Bethyl Laboratories Inc.	A120-201P; RRID: AB_67265	1:10000		
Goat anti-rabbit IgG-HRP antibody	Santa Cruz Biotechnology	sc-2054; RRID: AB_631748	1:2000		
Donkey anti-Mouse IgG (H+L) Highly Cross-Adsorbed Secondary Antibody, Alexa Fluor 488	Thermo Fisher Scientific	A-21202; RRID: AB_141607		1:1000	
Goat anti-Rabbit IgG (H+L) Highly Cross-Adsorbed Secondary Antibody, Alexa Fluor 488	Thermo Fisher Scientific	A-11034; RRID: AB_2576217		1:1000	
Goat anti-Mouse IgG (H+L) Highly Cross-Adsorbed Secondary Antibody, Alexa Fluor 568	Thermo Fisher Scientific	A-11031; RRID: AB_144696		1:1000	
Goat anti-Rabbit IgG (H+L) Cross-Adsorbed Secondary Antibody, Alexa Fluor 568	Thermo Fisher Scientific	A-11011; RRID: AB_143157		1:1000	
Goat anti-Mouse IgG (H+L) Highly Cross-Adsorbed Secondary Antibody, Alexa Fluor Plus 647	Thermo Fisher Scientific	A32728; RRID: AB_2633277		1:1000	

Supplementary Table 4. List of the reagents used in the present study.

Reagent	Source	Identifier
**Chemicals, Inhibitors and recombinant proteins**		
Blebbistatin	Sigma	B0560
Brefeldin A	Sigma	B7651
Cobalt(II) chloride	Sigma	60818
DL-Sulforaphane	Sigma	S4441
VEGF	Sigma	V7259
Prima1-Met	Cayman Chemical Company	900048
human TNFα	Invitrogen	PHC3015
Phenylmethanesulfonyl fluoride (PMSF)	Sigma	78830
Sodium fluoride (NaF)	Sigma	S-6521
Sodium orthovanadate (Na_3VO_4)	Sigma	S6508
Paclitaxel	Sigma	T7191
Protease Inhibitor Cocktail (CLAP)	Sigma	P8340
EasyTag™ EXPRESS35S Protein Labeling Mix, [35S]-, 2mCi	Perkin Elmer	NEG772002MC
Trichloroacetic acid	Sigma	T6399
D-biotin	Sigma	B4501
Fluorescein isothiocyanate-dextran 70 kDa	Sigma	FD70S
Crystal Violet	Sigma	C6158
Paraformaldehyde	Sigma	158127
**Commercial KIT**		
AccuPrime™ Taq DNA Polymerase High Fidelity	Invitrogen	12346086
Duolink In Situ Red Starter Kit Mouse/Rabbit	Sigma	DUO92101
TaqMan™ Mutation Detection Assay (castPCR)	Life Technologies	4465807
Picro Sirius Red Stain Kit (Connective Tissue Stain)	Abcam	ab150681
QIAamp DNA FFPE Tissue Kit	Qiagen	56404
Quantitect reverse transcription kit	Qiagen	205310
SsoAdvanced™SYBR® Green Master Mix	Biorad	1725270
miScript II RT Kit	Qiagen	218160
miScript SYBR® Green PCR Kit	Qiagen	218073
**Experimental Model: Cell Lines**		
BJ-EHT-ER-RAS	Prof. Reuven Agami, NKI-AVL, Amsterdam, Netherlands	Voorhoeve and Agami, 2003
DU 145	Prof. Giovanni Blandino, Cancer Institute "Regina Elena", Rome, Italy	ATCC HTB-81
H1299	ATCC	ATCC CRL-5803
HBL-100	ATCC	ATCC HTB-124
HEK-293GP	Prof. Stefano Piccolo, Università degli Studi di Padova, Padua, Italy	CVCL_E072
HEK-293T	Prof. Enzo Medico, Candiolo Cancer Institute – IRCSS, Candiolo (TO), Italy	ATCC CRL-3216
HT-29	Prof. Giovanni Blandino, Cancer Institute "Regina Elena", Rome, Italy	ATCC HTB-38
HUVEC	Prof. Roberta Bulla, Università degli Studi di Trieste, Trieste, Italy	Maruyama, 1963
LFS fibroblasts	Dr. David Malkin, Genetics and Genome Biology Program, The	

	Hospital for Sick Children, Toronto, ON. Canada	
Mahlavu	Prof. Pierre Hainaut, IAB, Grenoble, France	CVCL_045
MCF7		
MCF 10A	Prof. Stefano Piccolo, Università degli Studi di Padova, Padua, Italy	ATCC CRL-10317
MDA-MB-231	Prof. Stefano Piccolo, Università degli Studi di Padova, Padua, Italy	ATCC HTB-26
MDA-MB-468	Prof. Giovanni Blandino, Cancer Institute “Regina Elena”, Rome, Italy	ATCC HTB-132
SK-BR-3	Prof. Stefan Schoeftner, LNCIB, Trieste, Italy	ATCC HTB-30
SUM-159PT	Prof. Giovanni Blandino, Cancer Institute “Regina Elena”, Rome, Italy	CVCL_5423
TOV-112D	ATCC	ATCC CRL-11731
**Experimental Model: Mouse**		
NOD/SCID common γ chain knockout	NSG, Charles River	
**Molecular probes**		
Hoechst 33342	Thermo Fisher Scientific	H1399
Phalloidin-Alexa Fluor 488	Invitrogen	A12379
Lectin HPA from *Helix pomatia*, Alexa Fluor™ 488 Conjugate	Thermo Fisher Scientific	L11271
FITC-Dextran (avg. m.w. 70 kDa)	Sigma-Aldritch	FD70S
**Software**		
Fiji ImageJ (version 2.0-rc-69/1.52p)	Schindelin, J.; Arganda-Carreras, I. & Frise, E. et al. (2012), Nature Methods	https://fiji.sc/
GraphPad 8	GraphPad Software	https://www.graphpad.com/scientific-software/prism/
Volocity 3D	Perkin Elmer	http://www.perkinelmer.com/volocity
R (version 3.5.1)	R Development Core Team (2019)	https://www.r-project.org/
Gene Expression Module (GSGX) Version 1.9		
CFX Maestro™ 1.1		
EzC13.91		
NIS- Elements 4.6		
MaxQuant		www.maxquant.org

Supplementary References

1. Montagner, M. *et al.* SHARP1 suppresses breast cancer metastasis by promoting degradation of hypoxia-inducible factors. *Nature* **487**, 380–384 (2012).

2. Pereira, B. *et al.* The somatic mutation profiles of 2,433 breast cancers refines their genomic and transcriptomic landscapes. *Nat. Commun.* **7**, (2016).

3. Deshmukh, A. S., Cox, J., Jensen, L. J., Meissner, F. & Mann, M. Secretome analysis of lipid-induced insulin resistance in skeletal muscle cells by a combined experimental and bioinformatics workflow. *J. Proteome Res.* **14**, 4885–4895 (2015).

4. Carbon, S. *et al.* The Gene Ontology Resource: 20 years and still GOing strong. *Nucleic Acids Res.* **47**, D330–D338 (2019).

5. Ashburner, M. *et al.* The Gene Ontology Consortium, Michael Ashburner1, Catherine A. Ball3, Judith A. Blake4, David Botstein3, Heather Butler1, J. Michael Cherry3, Allan P. Davis4, Kara Dolinski3,

Selina S. Dwight3, Janan T. Eppig4, Midori A. Harris3, David P. Hill4, Laurie Is. *Nat. Genet.* **25**, 25–29 (2000).

6. Bendtsen, J. D., Jensen, L. J., Blom, N., Von Heijne, G. & Brunak, S. Feature-based prediction of non-classical and leaderless protein secretion. *Protein Eng. Des. Sel.* **17**, 349–356 (2004).

7. Almagro Armenteros, J. J. *et al.* SignalP 5.0 improves signal peptide predictions using deep neural networks. *Nat. Biotechnol.* **37**, 420–423 (2019).

8. Bateman, A. UniProt: A worldwide hub of protein knowledge. *Nucleic Acids Res.* **47**, D506–D515 (2019).

UNCROPPED FIG1

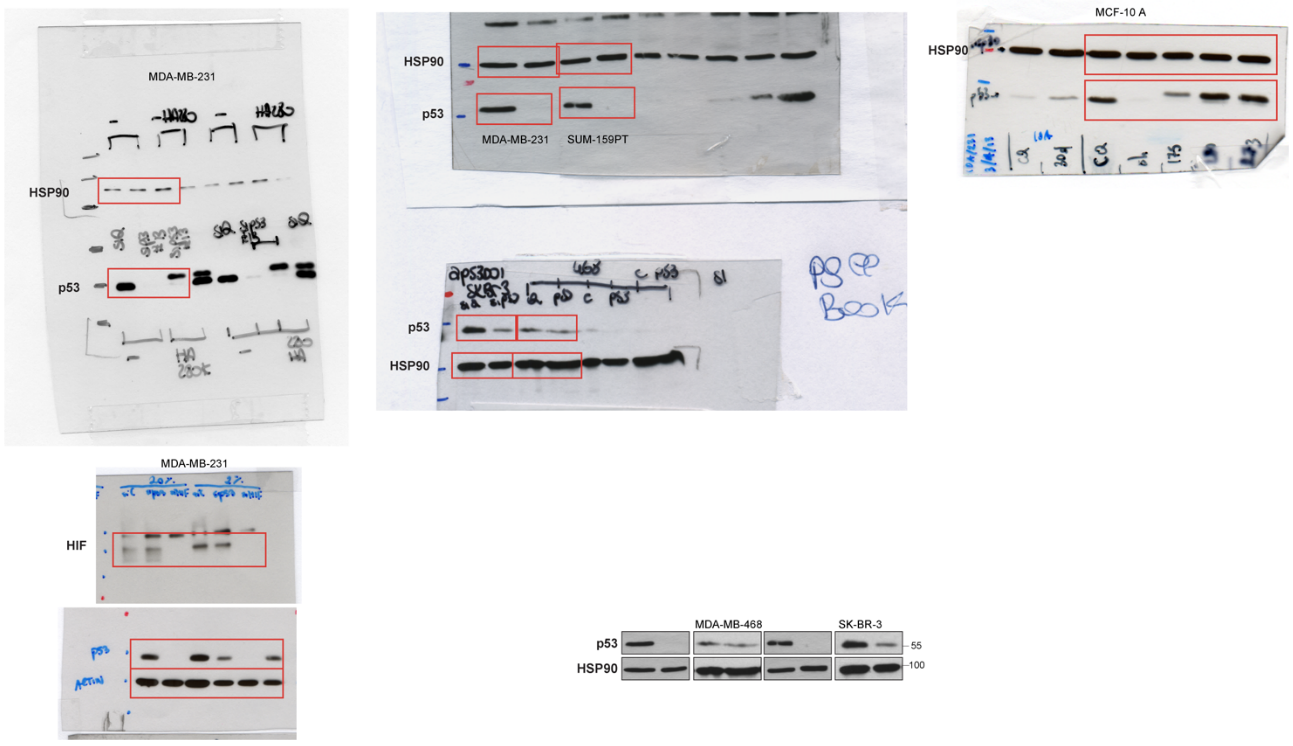

UNCROPPED FIG2

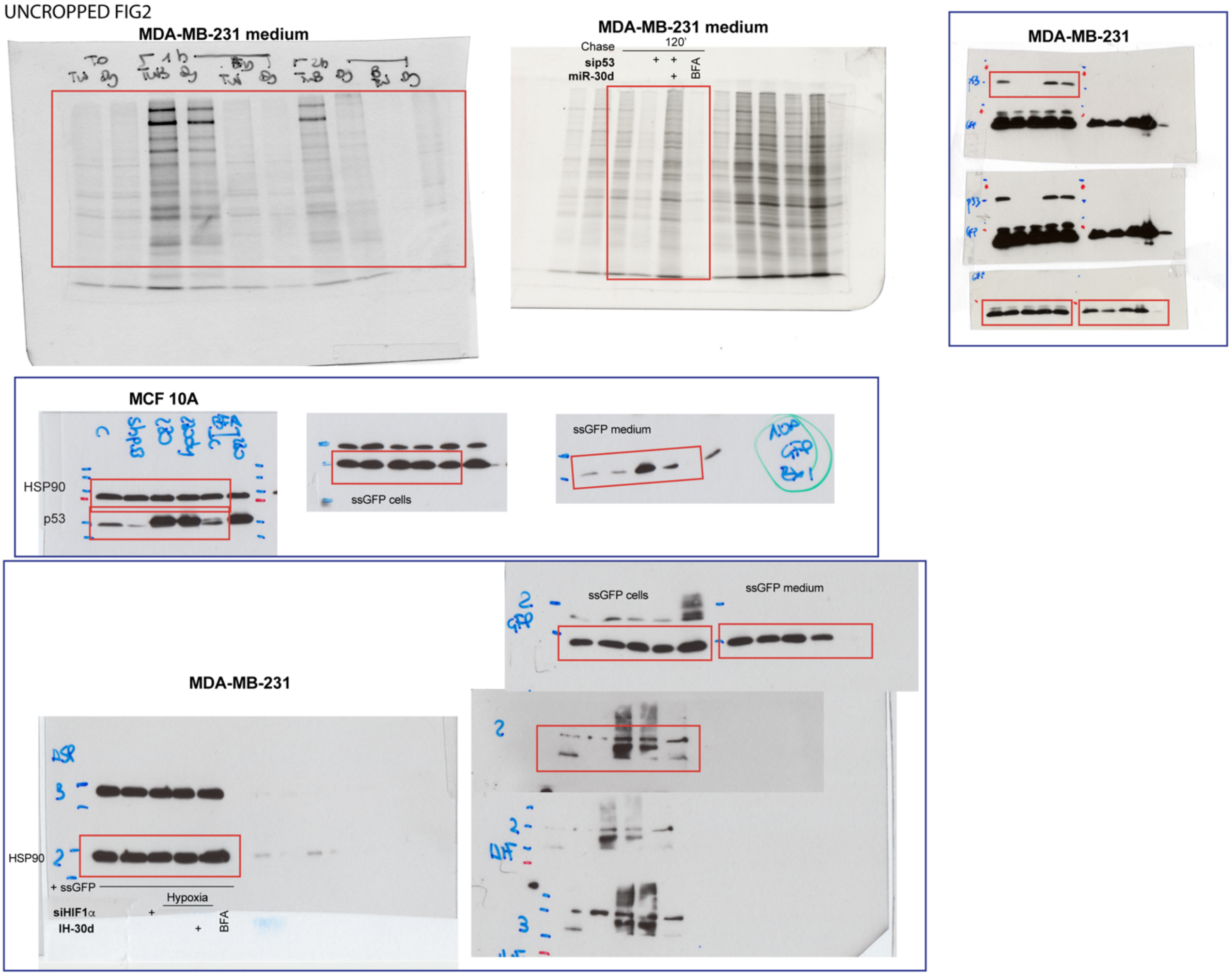

UNCROPPED FIG4

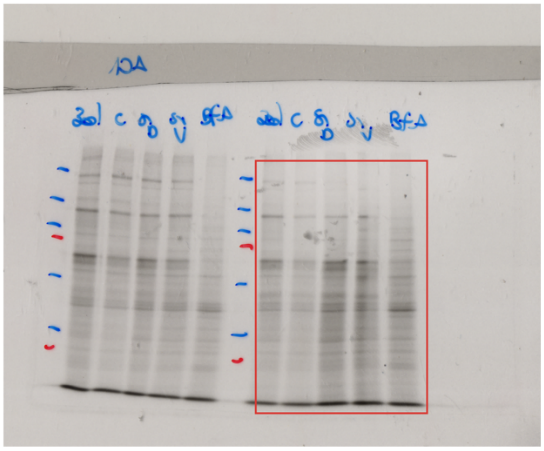

UNCROPPED FIG5

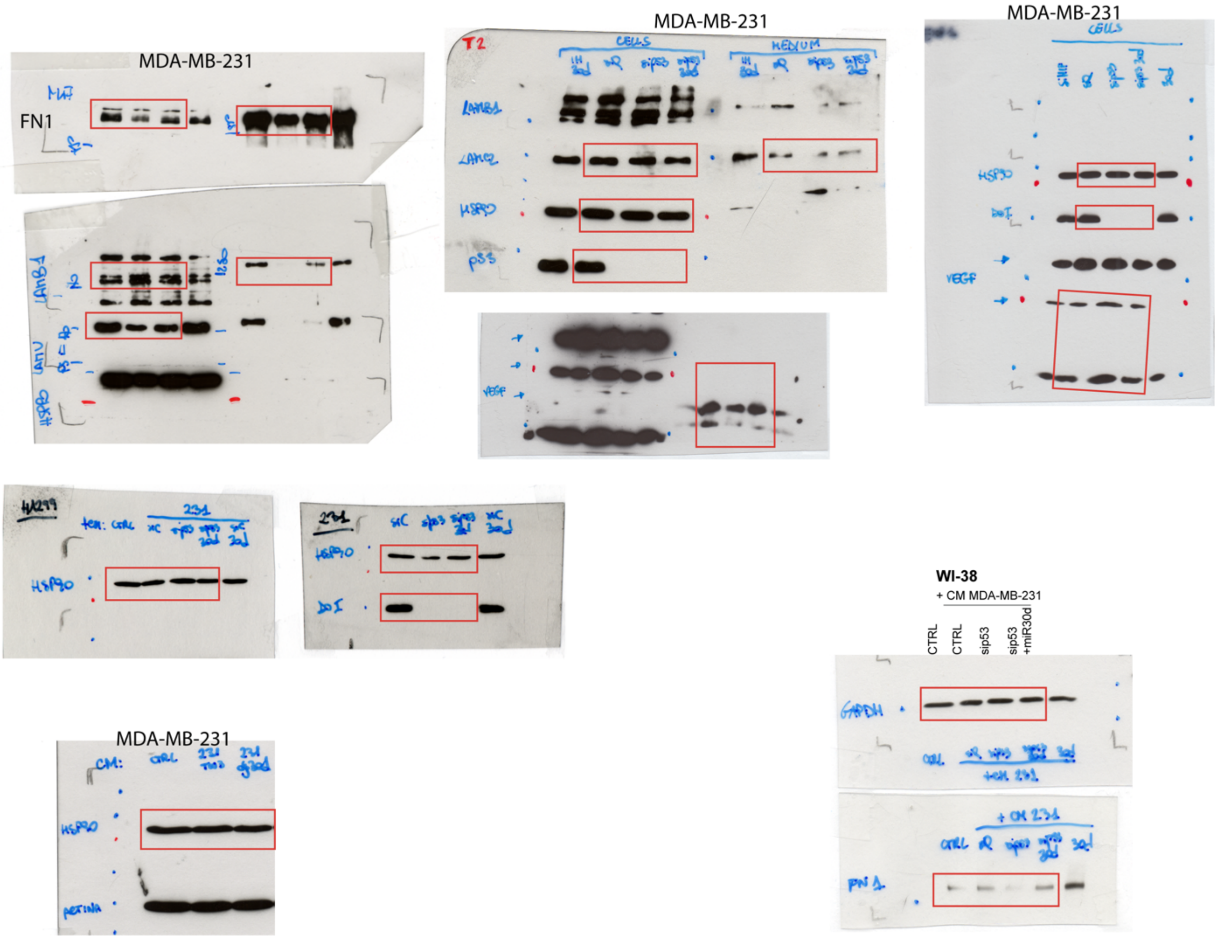

UNCROPPED FIG7

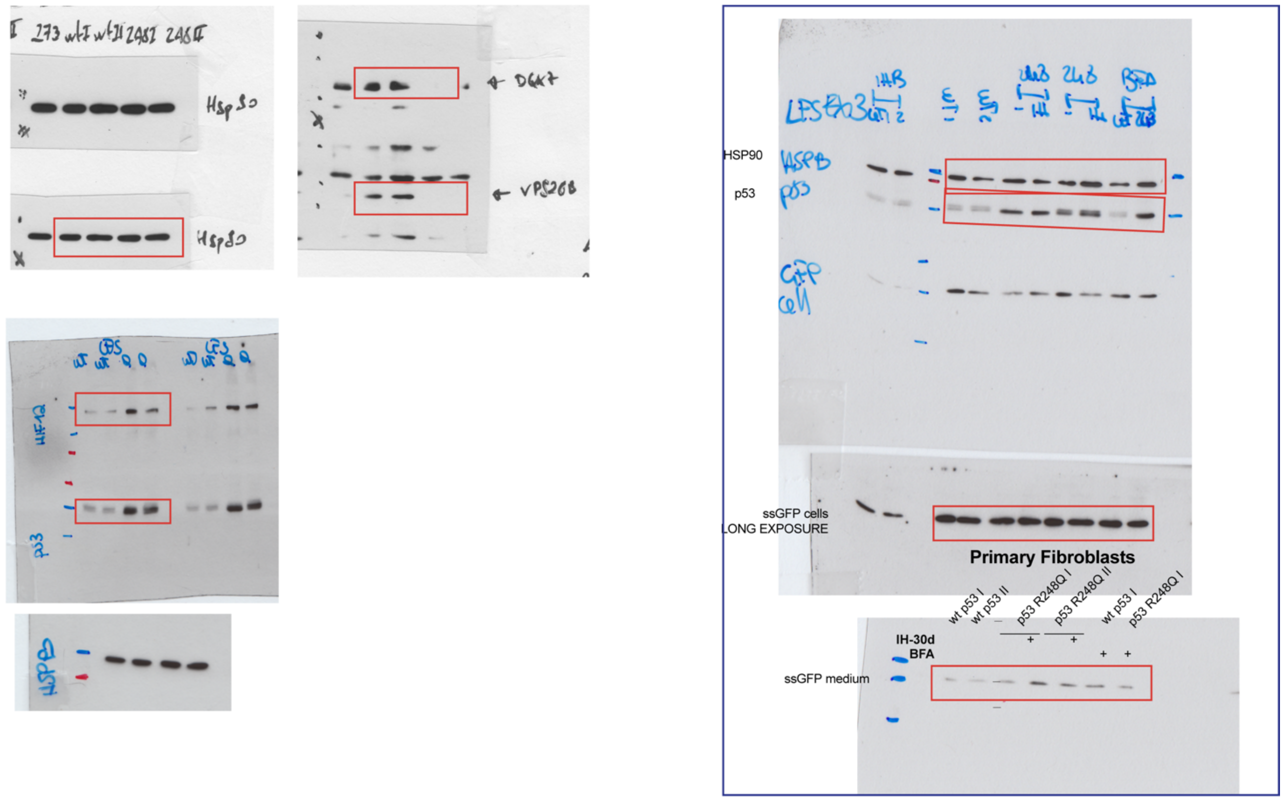

UNCROPPED SUPPL FIG1

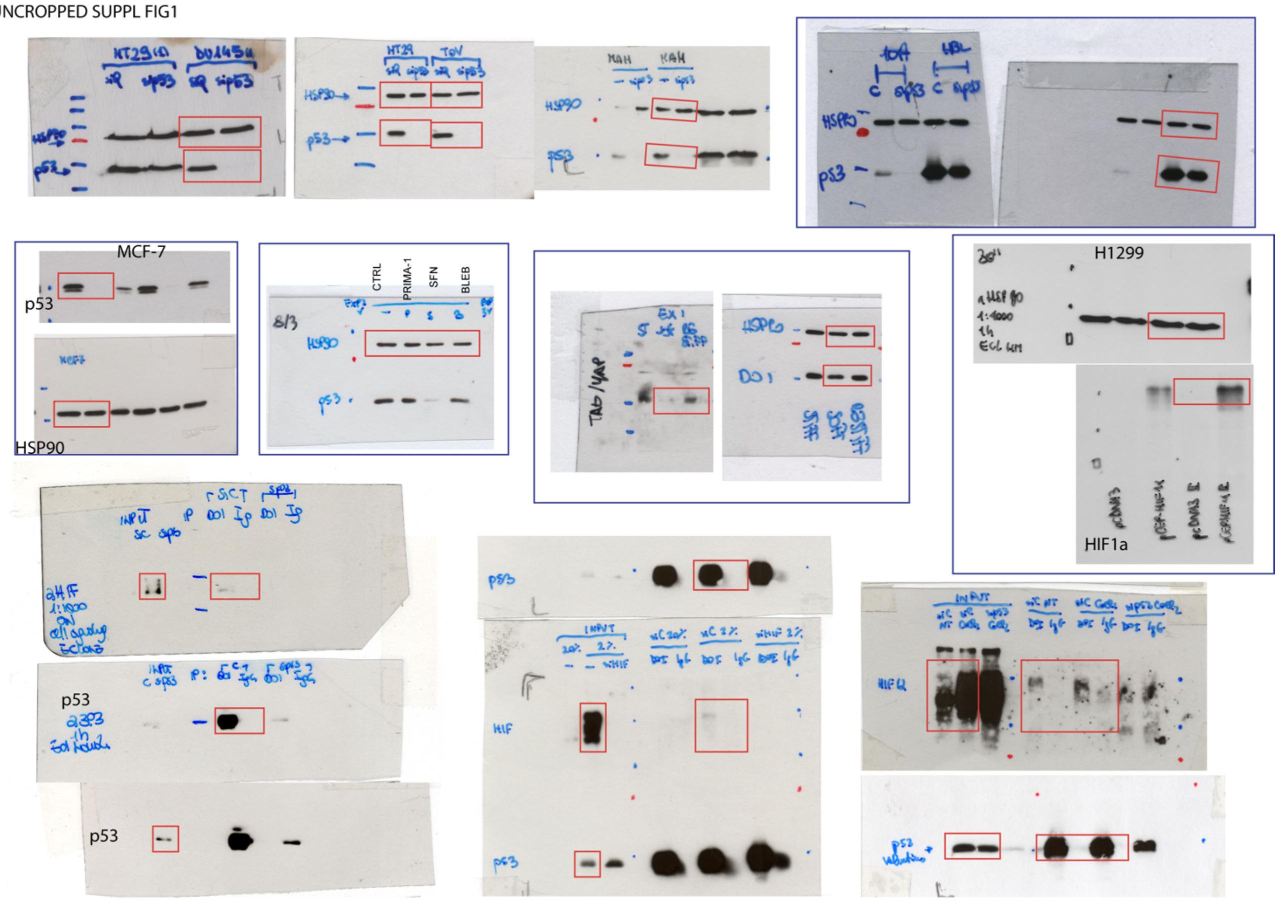

UNCROPPED SUPPL FIG3

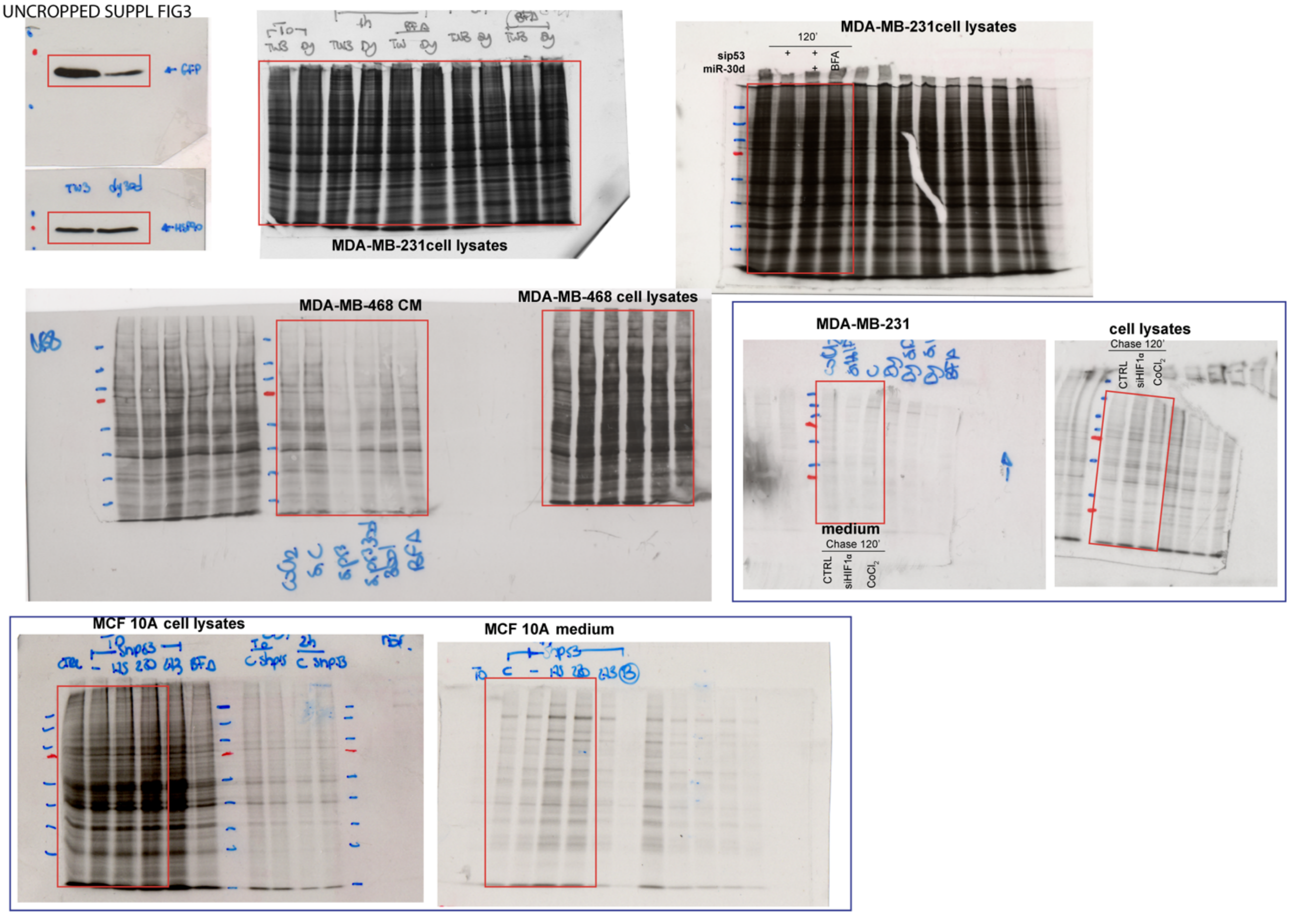

UNCROPPED SUPPL FIG4

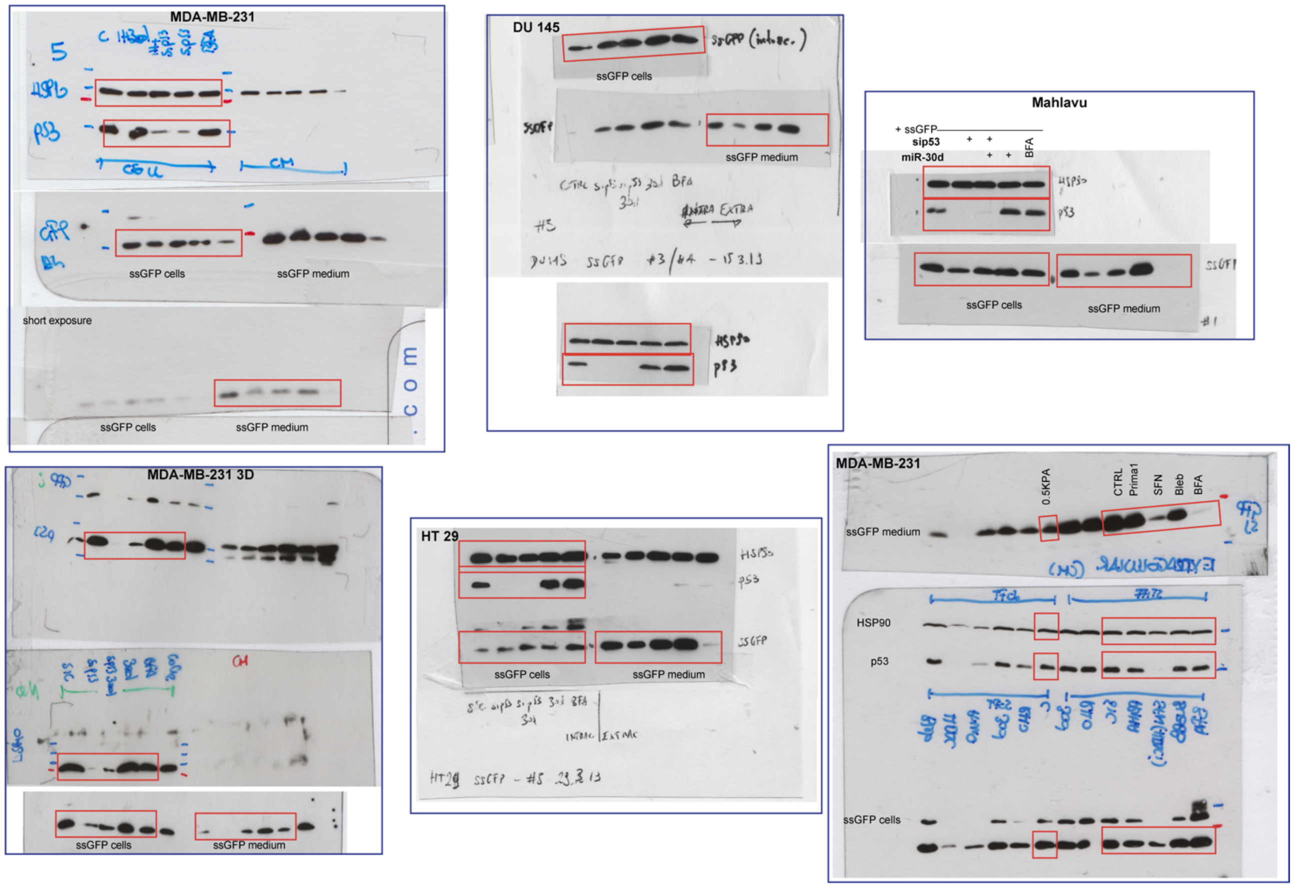

UNCROPPED SUPPL FIG5

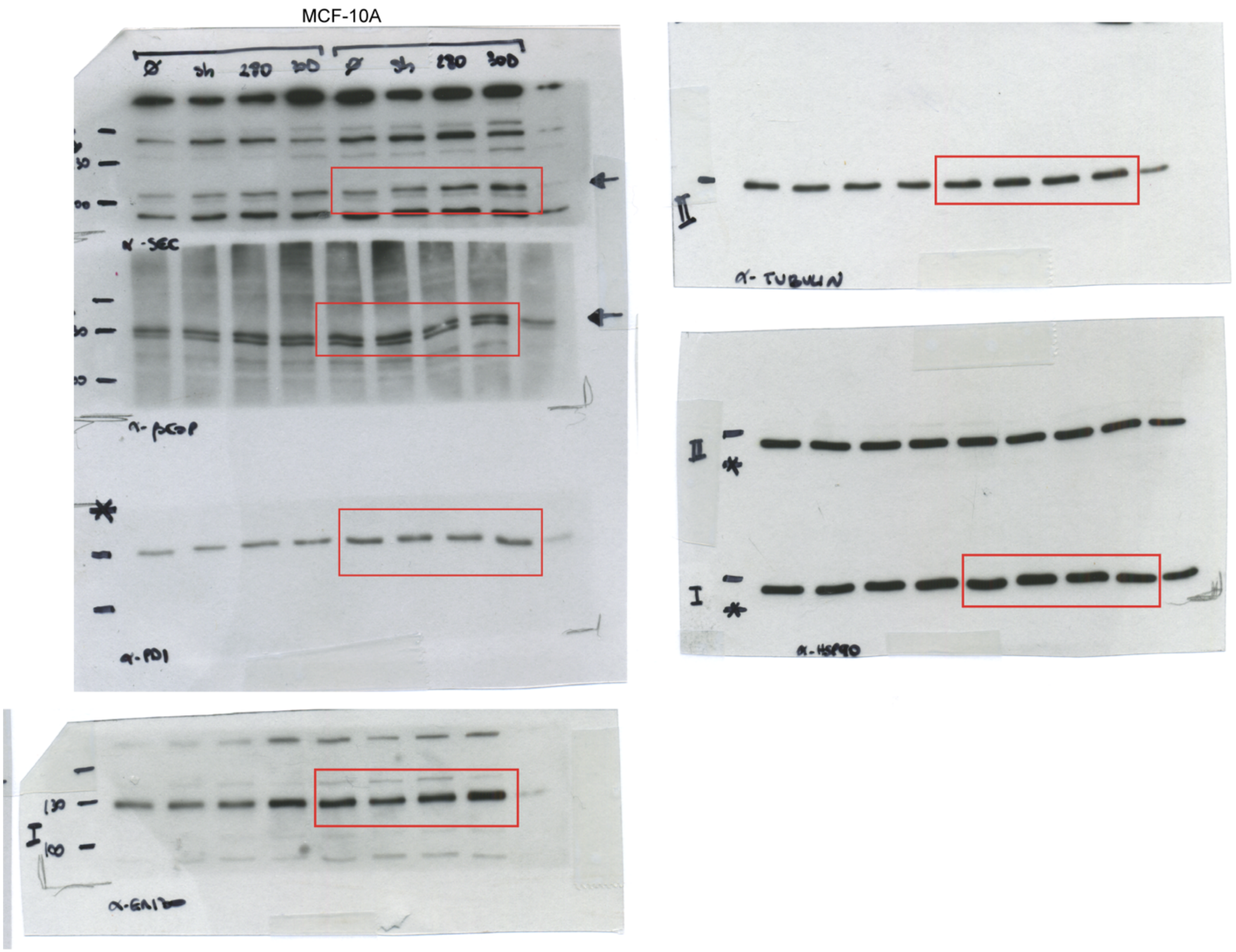

UNCROPPED SUPPL FIG6

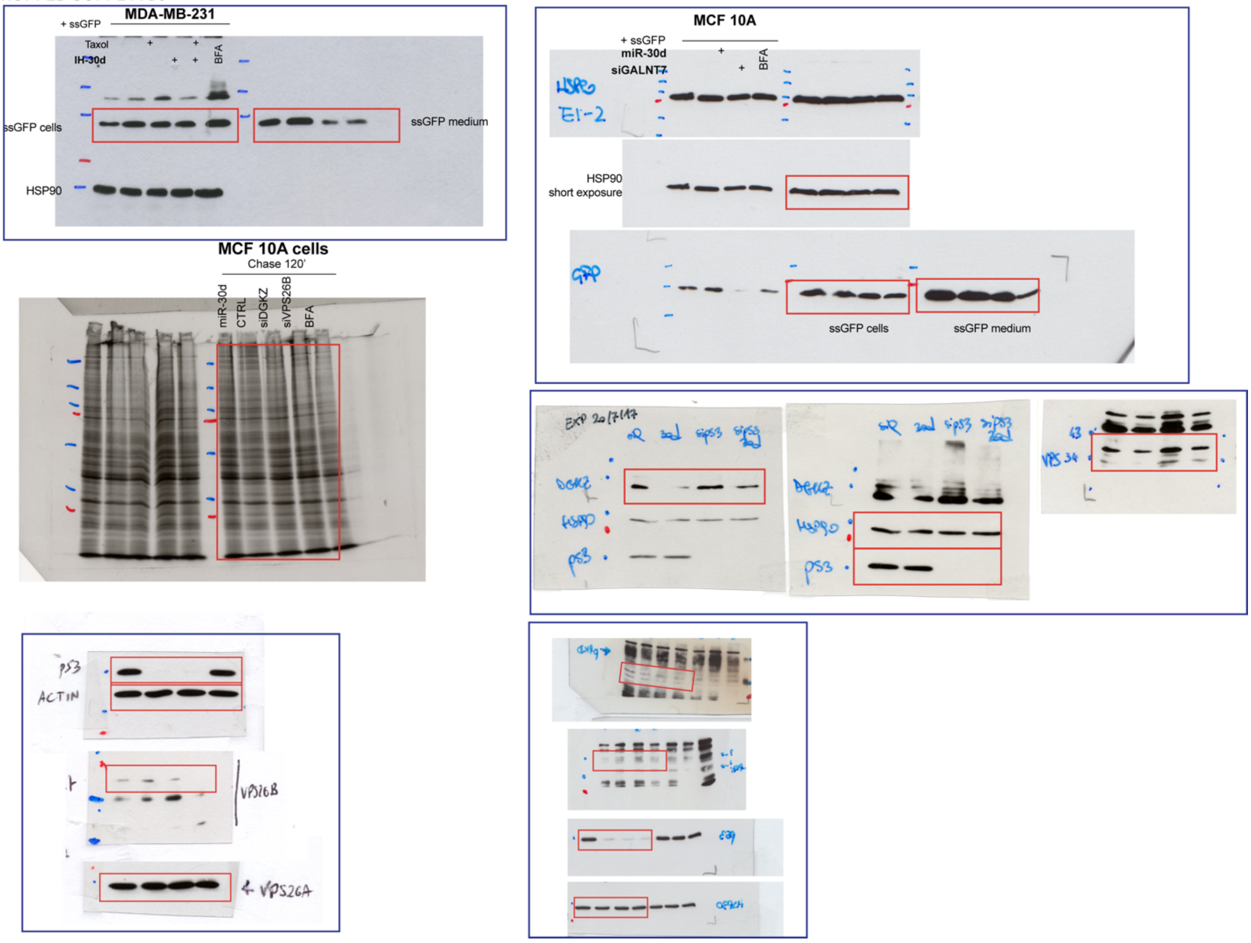

UNCROPPED SUPPL FIG8

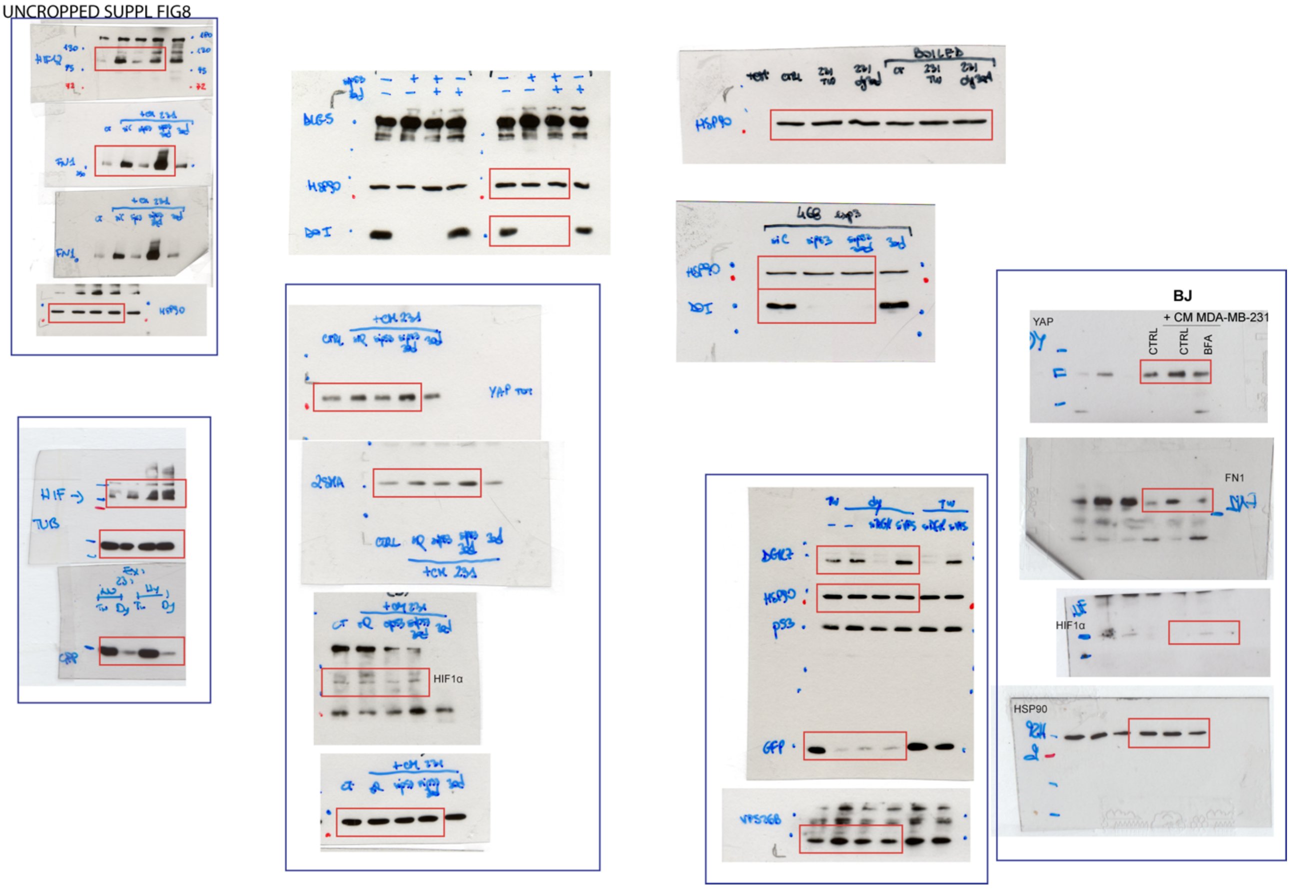

UNCROPPED SUPPL FIG9

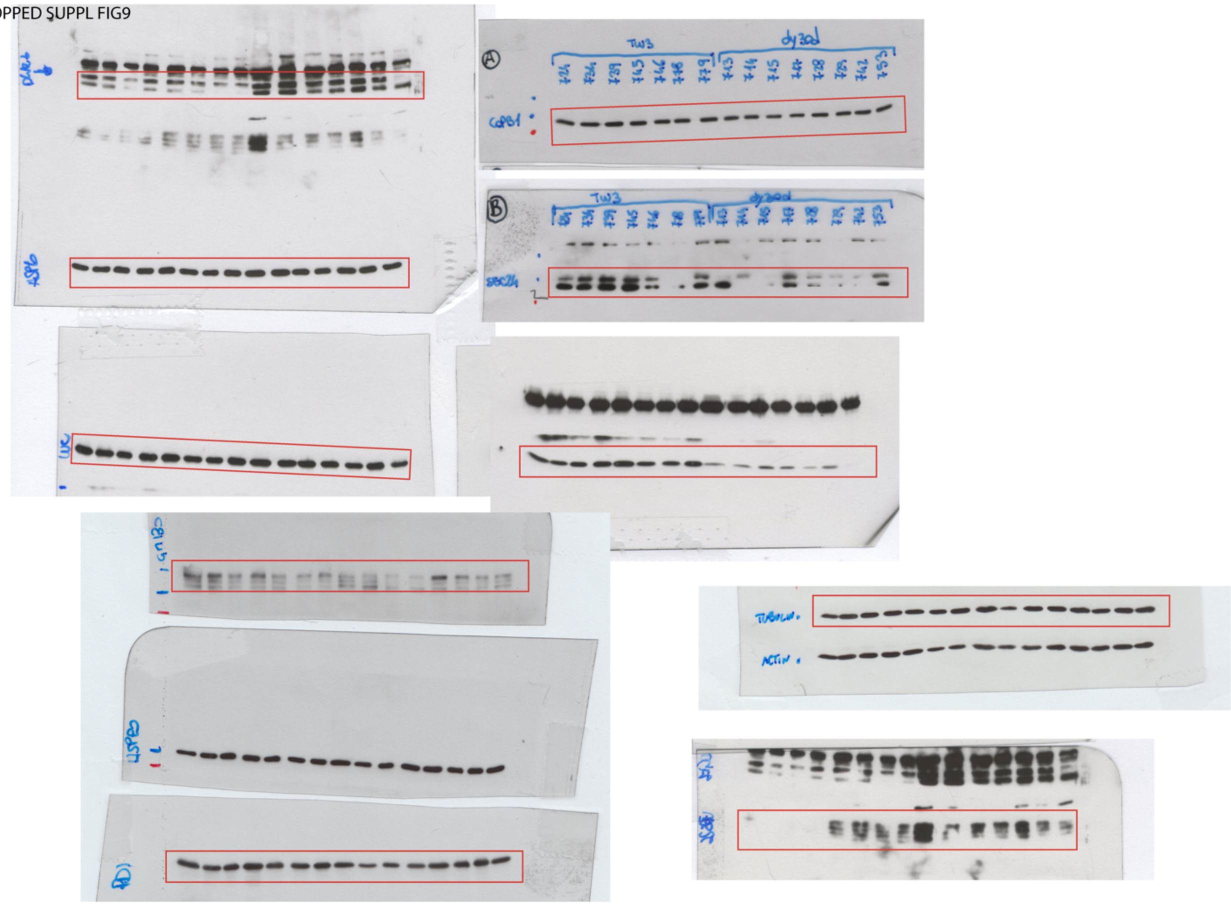

Supplement: Supplementary file 1 — Supplementary Information [file 41467_2020_17596_MOESM1_ESM.pdf]
