# Supplementary material for: Mutant p53 induces Golgi tubulo-vesiculation driving a prometastatic secretome
Source: Nat Commun. 2020 Aug 7;11:3945. doi: 10.1038/s41467-020-17596-5 (PMC7414119; doi:10.1038/s41467-020-17596-5)
Supplement: Supplementary file 3 — Description of Additional Supplementary Files [file 41467_2020_17596_MOESM3_ESM.docx]

Description of Additional Supplementary Files

**Title:** Supplementary Movie1. Z-stack reconstitution of Golgi Apparatus (GM130 staining) in MCF 10A

cells, either in control cells or upon miR-30d overexpression. Scale bar, 20 µm.

**Title:** Supplementary Movie2. Live-imaging movie showing the cellular localization of the RUSH

reporter MannII-SBP-EGFP after addition of biotin in MCF 10A cells transfected with either CTRL

or miR30d mimic. Scale bar, 20 µm. Total time 2 hours.

**Title:** Supplementary Data file1. Gene Ontology (GO) enrichment analysis of genes differentially

expressed in MDA-MB-231 cells transduced with miR-30d decoy as compared to controltransduced cells, using the DAVID database (GO TERM Functional Analysis).

**Title:** Supplementary Data file 2. Lists of differentially secreted proteins by mut-p53/miR-30d axis.

Official gene symbol is indicated and Gene Ontology (GO) enrichment analysis of proteins

differentially secreted by MDA-MB-231 cells (from data shown in Fig. 2G), using the DAVID

database (GO TERM Functional Analysis).
