# Supplementary material for: Mutant p53 induces Golgi tubulo-vesiculation driving a prometastatic secretome
Source: Nat Commun. 2020 Aug 7;11:3945. doi: 10.1038/s41467-020-17596-5 (PMC7414119; doi:10.1038/s41467-020-17596-5)
Supplement: Supplementary file 8 — Reporting Summary [file 41467_2020_17596_MOESM8_ESM.pdf]

nature research

Corresponding author(s): Giannino Del Sal

Last updated by author(s): 2020/06/26

Reporting Summary

Nature Research wishes to improve the reproducibility of the work that we publish. This form provides structure for consistency and transparency in reporting. For further information on Nature Research policies, see our Editorial Policies and the Editorial Policy Checklist.

Statistics

For all statistical analyses, confirm that the following items are present in the figure legend, table legend, main text, or Methods section.

n/a	Confirmed	
☐	☒	The exact sample size (*n*) for each experimental group/condition, given as a discrete number and unit of measurement
☐	☒	A statement on whether measurements were taken from distinct samples or whether the same sample was measured repeatedly
☐	☒	The statistical test(s) used AND whether they are one- or two-sided<br>*Only common tests should be described solely by name; describe more complex techniques in the Methods section.*
☒	☐	A description of all covariates tested
☐	☒	A description of any assumptions or corrections, such as tests of normality and adjustment for multiple comparisons
☐	☒	A full description of the statistical parameters including central tendency (e.g. means) or other basic estimates (e.g. regression coefficient) AND variation (e.g. standard deviation) or associated estimates of uncertainty (e.g. confidence intervals)
☐	☒	For null hypothesis testing, the test statistic (e.g. *F*, *t*, *r*) with confidence intervals, effect sizes, degrees of freedom and *P* value noted<br>*Give P values as exact values whenever suitable.*
☒	☐	For Bayesian analysis, information on the choice of priors and Markov chain Monte Carlo settings
☒	☐	For hierarchical and complex designs, identification of the appropriate level for tests and full reporting of outcomes
☒	☐	Estimates of effect sizes (e.g. Cohen's *d*, Pearson's *r*), indicating how they were calculated

Our web collection on statistics for biologists contains articles on many of the points above.

Software and code

Policy information about availability of computer code

Data collection	Gene Expression Module (GSGX) Version 1.9<br>CFX Maestro™ 1.1<br>EzC13.91<br>NIS- Elements 4.6<br>MaxQuant (https://www.maxquant.org/)<br>Zen 2.0 imaging software
Data analysis	Fiji ImageJ (version 2.0-rc-69/1.52p) Schindelin, J.; Arganda-Carreras, I. & Frise, E. et al. (2012), Nature Methods https://fiji.sc/<br>GraphPad 8 GraphPad Software https://www.graphpad.com/scientific-software/prism/<br>Volocity 3D Perkin Elmer http://www.perkinelmer.com/volocity<br>R (version 3.5.1) R Development Core Team (2019) https://www.r-project.org/

For manuscripts utilizing custom algorithms or software that are central to the research but not yet described in published literature, software must be made available to editors and reviewers. We strongly encourage code deposition in a community repository (e.g. GitHub). See the Nature Research guidelines for submitting code & software for further information.

Data

Policy information about availability of data

All manuscripts must include a data availability statement. This statement should provide the following information, where applicable:

- Accession codes, unique identifiers, or web links for publicly available datasets
- A list of figures that have associated raw data
- A description of any restrictions on data availability

The authors declare that the data supporting findings of this study are available within the paper and its supplementary information files.
Array data that support the findings of this study have been deposited to GEO, accession number GSE133410.
Lists of differentially secreted proteins by mut-p53/miR-30d axis in MDA-MB-231 cells are reported in Supplementary Data file 2.
Public available data used in this were obtained from : Ensembl (http://www.ensembl.org/index.html); UCSC (http://genome.ucsc.edu/cgibin/ hgGateway); DAVID Bioinformatics Resources 6.8 (https://david.ncifcrf.gov/) ; GSEA Gene Set Enrichment Analysis (https://www.gsea-msigdb.org/gsea/index.jsp); TargetScan (www.targetscan.org) ; GOCC (http://geneontology.org/); SecretomeP 2.0 (http://www.cbs.dtu.dk/services/SecretomeP/); SignalIP (http://www.cbs.dtu.dk/services/SignalP/); UniProt (https://www.uniprot.org/);
Molecular Taxonomy of Breast Cancer International Consortium, METABRIC27 ; The Cancer Genome Atlas (TCGA) breast cancer dataset (https://www.cancer.gov/about-nci/organization/ccg/research/structural-genomics/tcga) .

Field-specific reporting

Please select the one below that is the best fit for your research. If you are not sure, read the appropriate sections before making your selection.

[x] Life sciences [ ] Behavioural & social sciences [ ] Ecological, evolutionary & environmental sciences

For a reference copy of the document with all sections, see nature.com/documents/nr-reporting-summary-flat.pdf

Life sciences study design

All studies must disclose on these points even when the disclosure is negative.

Sample size	The sample size was chosen to include at least three biological replicates and no statistical method was used to determine sample size.
Data exclusions	No data were excluded from the analysis.
Replication	Experiments were performed succesfully at least three times. All replication attempts were succesfull.
Randomization	For Animal study, animals were randomly put into cages and randomly assigned to experimental groups.<br>For experiment involving cellular and biological study three indipendent experiments have been perfomed, allocating randomly cells in experimental groups.
Blinding	For most of the other experiments the results were quantified and appropriate statistical tests were performed to evaluate difference and statistical significance. When blinding was possible the analysis have been blinded.

Reporting for specific materials, systems and methods

We require information from authors about some types of materials, experimental systems and methods used in many studies. Here, indicate whether each material, system or method listed is relevant to your study. If you are not sure if a list item applies to your research, read the appropriate section before selecting a response.

Materials & experimental systems

n/a	Involved in the study	
[ ]	[x]	Antibodies
[ ]	[x]	Eukaryotic cell lines
[x]	[ ]	Palaeontology and archaeology
[ ]	[x]	Animals and other organisms
[ ]	[x]	Human research participants
[x]	[ ]	Clinical data
[x]	[ ]	Dual use research of concern

Methods

n/a	Involved in the study	
[x]	[ ]	ChIP-seq
[x]	[ ]	Flow cytometry
[x]	[ ]	MRI-based neuroimaging

Antibodies

Antibodies used	Actin Sigma-Aldrich A2066; RRID: AB_476693

CD31 Abcam ab124432; RRID:AB_2802125
COP Abcam ab2899; RRID: AB_2081300
COL6A2 Invitrogen PA5-65222; RRID:AB_2662555
DGKZ Sigma-Aldrich HPA051336; RRID: AB_2681448
Fibronectin (N1N2) GeneTex GTX112794; RRID: AB_1950298
FREM2 Abcam ab117612; RRID: AB_10933708
GAPDH (6C5) Santa Cruz Biotechnology sc-32233; RRID: AB_627679
Giantin Abcam ab80864; RRID: AB_10670397
GFP home-made rabbit polyclonal antibody N/A
GM130 BD 610822; RRID: AB_10015242
HIF-1 (D2U3T) Cell Signaling Technology #14179; RRID: AB_2622225
HIF-1(H1alpha67)
Novus Biologicals NB100-105; RRID:
AB_10001154
HSP90 alpha/beta (F-8) Santa Cruz Biotechnology sc13119; RRID: AB_675659
Ki67 (Sp6) Abcam ab16667; RRID: AB_302459
Laminin 5 (2 chain) Millipore MAB19562; RRID: AB_94454
Laminin-b1 Abcam ab69633; RRID: AB_1269284
Laminin beta-3 (CL3363) Invitrogen MA524655; RRID: AB_2637270
Luciferase Thermo Fisher Scientific pa1-46333; RRID: AB_1090406
p53 DO-1 Santa Cruz Biotechnology sc-126; RRID: AB_628082
p53 home-made rabbit polyclonal antibody N/A
p53 FL-393 Santa Cruz Biotechnology sc-6243; RRID: AB_653753
p53 (DO-7) Leica Biosystems NCL-L-p53-DO7; RRID: AB_563936
PDIA5 (RL90) Abcam ab2792; RRID: AB_303304
PKD/PKCμ (D4J1N) Cell Signaling Technology #90039; RRID: AB_2800149
Phospho-PKD Ser744/748 Cell Signaling Technology #2054; RRID: AB_2172539
SEC24A Abcam ab102660; RRID: AB_10711917
a-SMA Abcam ab5694; RRID: AB_2223021
Tubulin Sigma-Aldrich T5168; RRID: AB_477579
Acetylated-Tubulin Sigma-Aldrich T6793; RRID:
AB_477585
TGN46 Abcam ab50595; RRID: AB_2203289
VEGFA GeneTex GTX102643; RRID: AB_11174248
VPS26A Abcam ab23892; RRID: AB_2215043
VPS26B Sigma-Aldrich SAB4500246; RRID: AB_10743760
WWTR1 Sigma-Aldrich HPA007415; RRID: AB_1080602
YAP (H-125) Santa Cruz Biotechnology sc-15407; RRID: AB_2273277
Mouse normal IgG Santa Cruz Biotechnology sc-2025; RRID: AB_737182
Rabbit normal IgG Santa Cruz Biotechnology sc-2027; RRID: AB_737197
Mouse IgG-heavy and light chain cross-adsorbed antibody Bethyl Laboratories Inc. A90-516P; RRID: AB_10631212
Rabbit IgG-heavy and light chain cross-adsorbed antibody Bethyl Laboratories Inc. A120-201P; RRID: AB_67265
Goat anti-rabbit IgG-HRP antibody Santa Cruz Biotechnology sc-2054; RRID: AB_631748
Donkey anti-Mouse IgG (H+L) Highly Cross-Adsorbed Secondary Antibody, Alexa Fluor 488 Thermo Fisher Scientific A-21202; RRID: AB_141607
Goat anti-Rabbit IgG (H+L) Highly Cross-Adsorbed Secondary Antibody, Alexa Fluor 488 Thermo Fisher Scientific A-11034; RRID: AB_2576217
Goat anti-Mouse IgG (H+L) Highly Cross-Adsorbed Secondary Antibody, Alexa Fluor 568 Thermo Fisher Scientific A-11031; RRID: AB_144696
Goat anti-Rabbit IgG (H+L) Cross-Adsorbed Secondary Antibody, Alexa Fluor 568 Thermo Fisher Scientific A-11011; RRID: AB_143157
Goat anti-Mouse IgG (H+L) Highly Cross-Adsorbed Secondary Antibody, Alexa Fluor Plus 647 Thermo Fisher Scientific A32728; RRID: AB_2633277

Validation

Actin Sigma-Aldrich A2066; RRID: AB_476693 Reactivity:wide range, human, chicken, amoeba, slime mold, vertebrates
Validation: Anti-Actin specifically stains typical stress fibers in cultured chicken fibroblasts by manufacturer
CD31 Abcam ab124432; RRID:AB_2802125 Tested applications
Suitable for: WB, IHC-P
Positive controlWB: Mouse Heart and Spleen tissue lysates and TH2 whole cell lysate. IHC-P: Mouse Placenta tissue.by manufacturer

COP Abcam ab2899; RRID: AB_2081300 Suitable for: WB, ICC/IF, IP
Positive controlBALB/3T3 whole cell lysate (ab7901) can be used as a positive control in WB by manufacturer

COL6A2 Invitrogen PA5-65222; RRID:AB_2662555 Tested in Immunohistochemistry (IHC) applications. This antibody reacts with Human samples by manufacturer

DDGKZ Sigma-Aldrich HPA051336; RRID: AB_2681448 application(s) immunoblotting immunofluorescence immunohistochemistry . species reactivity human; developed and validated by the Human Protein Atlas (HPA) project

Fibronectin (N1N2) GeneTex GTX112794; RRID: AB_1950298 Application. WB, ICC/IF, IHC-P, IHC-Fr, IP, ELISA. Reactivity. Human, MouseValidated by manufacturer

FREM2 Abcam ab117612; RRID: AB_10933708 Tested applications WB, ELISA, IHC-P, ICC
Species reactivity Reacts with: Human Predicted to work with: Mouse
Positive control Human Breast and Human Kidney tissue
Validated by manufacturer
GAPDH (6C5) Santa Cruz Biotechnology sc-32233; RRID: AB_627679 recommended for detection of GAPDH of mouse, rat, human, rabbit and Xenopus origin by WB, IP and IF
Validated by manufacturer

Giantin Abcam ab80864; RRID: AB_10670397 Suitable for: IHC-P, ICC/IF, WB
Species reactivity Human
Positive control This antibody gave a positive signal in cervival tissue and MCF7 whole cells using immunohistochemistry and immunocytochemistry, respectively.
Validated by manufacturer

GFP home-made rabbit polyclonal antibody N/A Suitable for: WB, Immunofluorescence
Validated by overexpression
GM130 BD 610822; RRID: AB_10015242 ApplicationWestern blot (Routinely Tested)
Immunofluorescence (Tested During Development) by manufacturer

HIF-1alpha (D2U3T) Cell Signaling Technology #14179; RRID: AB_2622225 Applications: WB, ChIP, ChIP-seq
Reactivity:Human Mouse Rat Monkey
Validated by manufacturer
HIF-1(H1alpha67)
Novus Biologicals NB100-105; RRID:
AB_10001154 tested in 10 confirmed species: Human, Mouse, Rat, Bovine, Canine, Porcine, Primate, Rabbit, Sheep, Xenopus.
in literature reported 16 applications: ChIP, ELISA, EMSA, FLOW, GS, IA, IB, ICC/IF, IF, IHC, IHC-Fr, IHC-P, IHC/IF, IP, PLA, WB.
By manufacturer website

HSP90 alpha/beta (F-8) Santa Cruz Biotechnology sc13119; RRID: AB_675659 HSP 90α/β (F-8) is recommended for detection of HSP 90α and HSP 90β of mouse, rat and human origin by Western Blotting
Positive Controls: C6 whole cell lysate: sc-364373.

Ki67 (Sp6) Abcam ab16667; RRID: AB_302459 Tested applications
Suitable for: IHC-FoFr, ICC/IF, Flow Cyt, IHC-Fr, WB, IHC-P, mIHC
Species reactivity: Mouse, Rat, Human, Common marmoset
Knock Out testet by manufacturer

Laminin 5 (2 chain) Millipore MAB19562; RRID: AB_94454 Anti-Laminin-5 (γ2 chain) Antibody, clone D4B5 is an antibody against Laminin-5 (γ2 chain) for use in ELISA, IH(P) & WB.
by manufacturer : Evaluated by Western Blot on recombinant Laminin-5.
Control
Human pancreatic tumor tissue, human breast carcinoma tissue or A-431 whole cell lysate
Laminin-b1 Abcam ab69633; RRID: AB_1269284 Validated by manufacturer for: WB, ELISA, IHC-P, ICC/IF
Reacts with: Human
Laminin beta-3 (CL3363) Invitrogen MA524655; RRID: AB_2637270 Validated Applications by manufacturer: Immunohistochemistry, Western Blot
Luciferase Thermo Fisher Scientific pa1-46333; RRID: AB_1090406 No reference available
p53 DO-1 Santa Cruz Biotechnology sc-126; RRID: AB_628082 recommended for detection of wild type and mutant p53 under denaturing and non-denaturing conditions of mouse, rat and human origin by WB, IP, IF, IHC(P) and FCM by manufacturer

p53 home-made rabbit polyclonal antibody N/A Suitable for: WB, Immunofluorescence
Validated by Knockout mice
p53 FL-393 Santa Cruz Biotechnology sc-6243; RRID: AB_653753 p53 (FL-393) is recommended for detection of p53 of mouse, rat and human origin by Western Blotting, immunoprecipitation , immunofluorescence), immunohistochemistry, flow cytometry and solid phase ELISA, ChIP-
p53 (DO-7) Leica Biosystems NCL-L-p53-DO7; RRID: AB_563936 NCL-L-p53-DO7 is recommended for the detection of human p53 protein in normal and neoplastic tissues, as an adjunct to conventional histopathology using non-immunologic histochemical stains.
PDIA5 (RL90) Abcam ab2792; RRID: AB_303304 Suitable for: ICC/IF, Electron Microscopy, IHC-P, IHC-Fr, IP, WB, ELISA, Inhibition Assay, Flow Cyt
Reacts with: Mouse, Rat, Hamster, Dog, Human, Pig, Monkey, African green monkey
Positive control
rat liver
PKD/PKCμ (D4J1N) Cell Signaling Technology #90039; RRID: AB_2800149 Validated Applications by manufacturer: WB, IP
Reactivity:
Human Monkey
Species reactivity is determined by testing in at least one approved application (e.g., western blot).

Phospho-PKD Ser744/748 Cell Signaling Technology #2054; RRID: AB_2172539 Validated Applications by manufacturer: WB, IP
Reactivity:
Human Monkey Mouse Rat

SEC24A Abcam ab102660; RRID: AB_10711917 Validated Applications by manufacturer: WB, IHC-P, ICC/IF
Reacts with: Human
a-SMA Abcam ab5694; RRID: AB_2223021 Suitable for: IHC-FoFr, ICC/IF, WB, ELISA, IHC-P, IHC-Fr
Reacts with: Mouse, Rat, Chicken, Guinea pig, Cow, Dog, Human, Pig
Validated by manufacturer in IHC-P: Mouse intestine and mesentery tissue. Mouse mammary tissue

Tubulin Sigma-Aldrich T5168; RRID: AB_477579 Validated by manufacturer for immunochemical assays such as immunoblotting, solidphase RIA and immunohistochemistry.

Acetylated-Tubulin Sigma-Aldrich T6793; RRID:
AB_477585 Validated by manufacturer for the localization of acetylated tubulin using various immunochemical assays such as ELISA, immunoblot, dot blot, solid phase RIA, electron microscopy and immunohistochemistry.

TGN46 Abcam ab50595; RRID: AB_2203289 Validated by manufacturer for: WB, IP, ICC/IF
Reacts with: Human
Positive control in A549 cells
VEGFA GeneTex GTX102643; RRID: AB_11174248 Validated by manufacturer for WB, IHC-P
Reactivity Human, Mouse, Rat
Positive Control: HepG2 conditioned medium , *A549

VPS26A Abcam ab23892; RRID: AB_2215043 Validated by manufacturer for: WB, IHC-P, ICC/IF, IP
Knockout validated
Reacts with: Mouse, Rat, Human

VPS26B Sigma-Aldrich SAB4500246; RRID: AB_10743760 Validated by manufacturer for :ELISA; western blot
species reactivity: mouse, human

WWTR1 Sigma-Aldrich HPA007415; RRID: AB_1080602 Validated by manufacturer and associated peer-reviewed papers on manufacturer website for : WB; IF; IHC
species reactivity: mouse, human

YAP (H-125) Santa Cruz Biotechnology sc-15407
; RRID: AB_2273277 Validated by manufacturer for YAP detection in mouse, rat and human origin by WB, IP, IF; IHC; ELISA; ChIP applications. Tested in 293T Lysate: sc-124676, PC-3 cell lysate: sc-2220 or HeLa nuclear extract.

Eukaryotic cell lines

Policy information about cell lines

Cell line source(s)

-BJ-EHT-ER-RAS from Prof. Reuven Agami, NKI-AVL, Amsterdam, Netherlands Voorhoeve and Agami, 2003.
-DU 145 from Prof. Giovanni Blandino, Cancer Institute “Regina Elena”, Rome, Italy ATCC HTB-81; STR genotyping identification.
-H1299 from ATCC, ATCC CRL-5803 STR genotyping identification.
-HEK-293GP from Prof. Stefano Piccolo, Università degli Studi di Padova, Padua, Italy CVCL_E072 .
-HEK-293T from Prof. Enzo Medico, Candiolo Cancer Institute – IRCSS, Candiolo (TO), Italy, ATCC CRL-3216.
-HT-29 from Prof. Giovanni Blandino, Cancer Institute “Regina Elena”, Rome, Italy ATCC HTB-38.
-HUVEC from Prof. Roberta Bulla, Università degli Studi di Trieste, Trieste, Italy
-Primary LFS fibroblasts from Dr. David Malkin, Genetics and Genome Biology Program, The Hospital for Sick Children, Toronto, ON. Canada
-Mahlavu from Prof. Pierre Hainaut, IAB, Grenoble, France. CVCL_045
-MCF 10A from Prof. Stefano Piccolo, Università degli Studi di Padova, Padua, Italy ATCC CRL-10317; STR genotyping identification.
-MDA-MB-231 from Prof. Stefano Piccolo, Università degli Studi di Padova, Padua, Italy ATCC HTB-26; STR genotyping identification.
MDA-MB-468 from Prof. Giovanni Blandino, Cancer Institute “Regina Elena”, Rome, Italy. ATCC HTB-132; STR genotyping identification.
-SK-BR-3 from Prof. Stefan Schoeftner, LNCIB, Trieste, Italy. ATCC HTB-30; STR genotyping identification.
-SUM-159PT from Prof. Giovanni Blandino, Cancer Institute “Regina Elena”, Rome, Italy. CVCL_5423; STR genotyping identification.
-TOV-112D from ATCC. ATCC CRL-11731; STR genotyping identification.
-MCF-7 from ATCC; ATCC HTB-22; STR genotyping identification.

	-HBL-100, ATCC; ATCC HTB-124, STR genotyping identification.
Authentication	Cells were subjected to STR genotyping with PowerPlex 18D System and confirmed in their identity comparing the results to reference cell databases (DMSZ, ATCC and JCRB databases) as described before.
Mycoplasma contamination	All cell lines were tested for mycoplasma contamination with negative results.<br>only SUM-159PT; TOV-112D; HBL-100; DU 145 cell lines showed mycoplasma contamination by fluorescent dye detection.
Commonly misidentified lines<br>(See ICLAC register)	No commonly misidentified cell lines were used

Animals and other organisms

Policy information about studies involving animals; ARRIVE guidelines recommended for reporting animal research

Laboratory animals	Mouse model,NOD/SCID common γ chain knockout (NSG, Charles River), females, Six-to-eight week-old
Wild animals	The study did not involved wild animals
Field-collected samples	The study did not involved samples collected from the field
Ethics oversight	Procedures involving animals and their care were in conformity with national (D.L. 26/2014 and subsequent implementing circulars) and international (EU Directive 2010/63/EU for animal experiments) laws and policies, and the experimental protocol (Authorization n. 1143/2015-PR) was approved by the Ethical Committee of the University of Padua (CEASA) and by the Italian Ministry of Health.

Note that full information on the approval of the study protocol must also be provided in the manuscript.

Human research participants

Policy information about studies involving human research participants

Population characteristics	Human breast cancer tissue sections were classified according to the World Health Organization classification criteria of the Tumors of the Breast (2013).
Recruitment	Human breast cancer tissue sections were selected from the archival samples of the Tumor Immunology Laboratory, Human Pathology Section, of the Department of Health Sciences, University of Palermo.<br>Human Primary Fibroblasts were obtained by skin biopsy samples collected at The Hospital for Sick Children, Toronto, Ontario, Canada Institutional Research (TP53 mutant, n = 2; TP53 wild-type, n = 2).
Ethics oversight	Samples were collected in accordance with the Helsinki Declaration, and the study was approved by the University of Palermo Ethical Review Board (approval number 09/2018).<br>For the use of Human Primary Fibroblasts informed consent was obtained from the patient or parent/legal guardian of the patient. For the use of primary patient samples for this research, the study was approved by the Hospital for Sick Children Research Ethics Board under the study title "Molecular characterization of Li-Fraumeni Syndrome and its variants"

Note that full information on the approval of the study protocol must also be provided in the manuscript.
