# Supplementary figures and images for: Mutant p53 induces Golgi tubulo-vesiculation driving a prometastatic secretome
Source: Nat Commun. 2020 Aug 7;11:3945. doi: 10.1038/s41467-020-17596-5 (PMC7414119; doi:10.1038/s41467-020-17596-5)

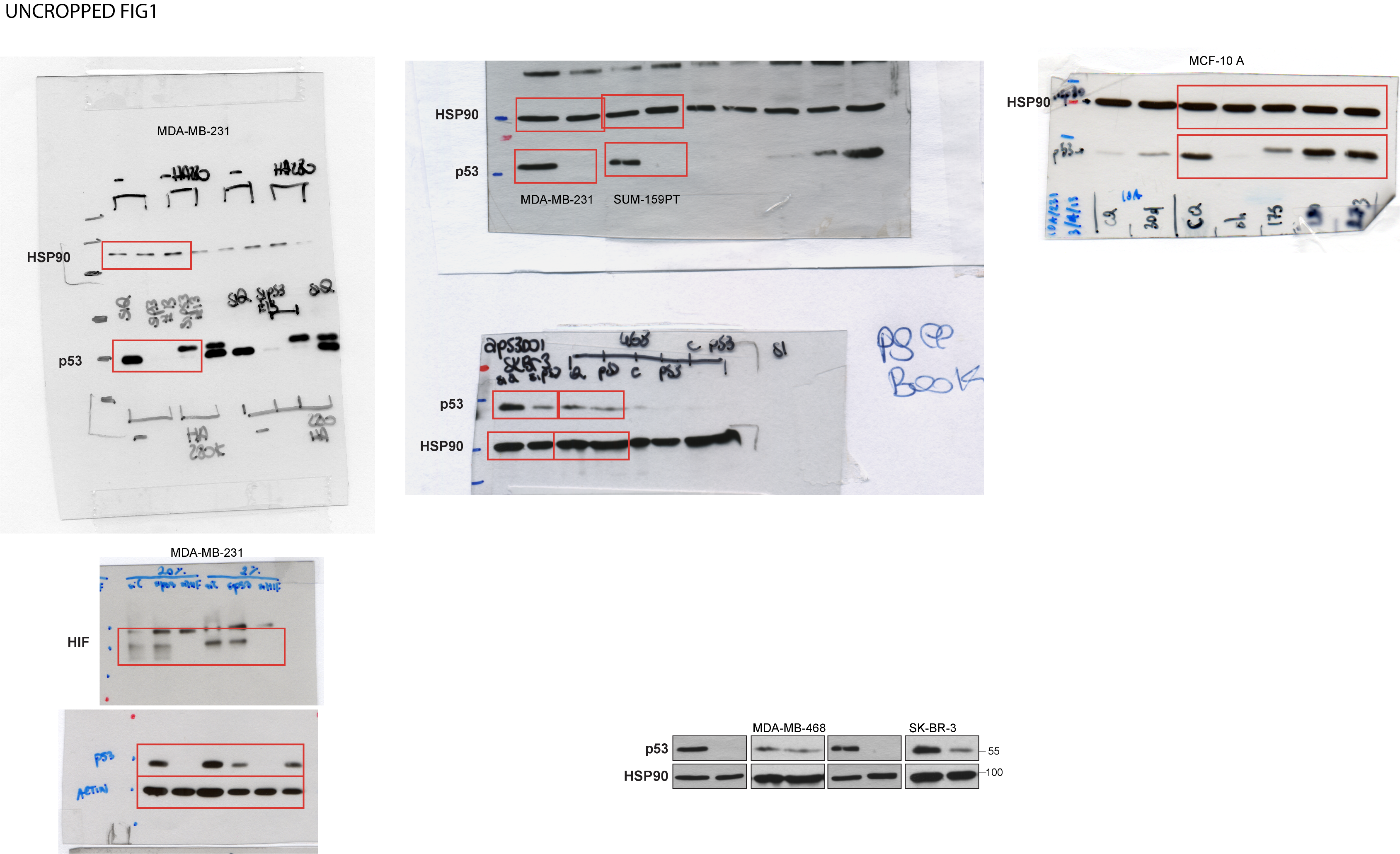

Supplement: Supplementary file 9 — Source Data File [file 41467_2020_17596_MOESM9_ESM.zip › cartella senza titolo/scan fig1.png]

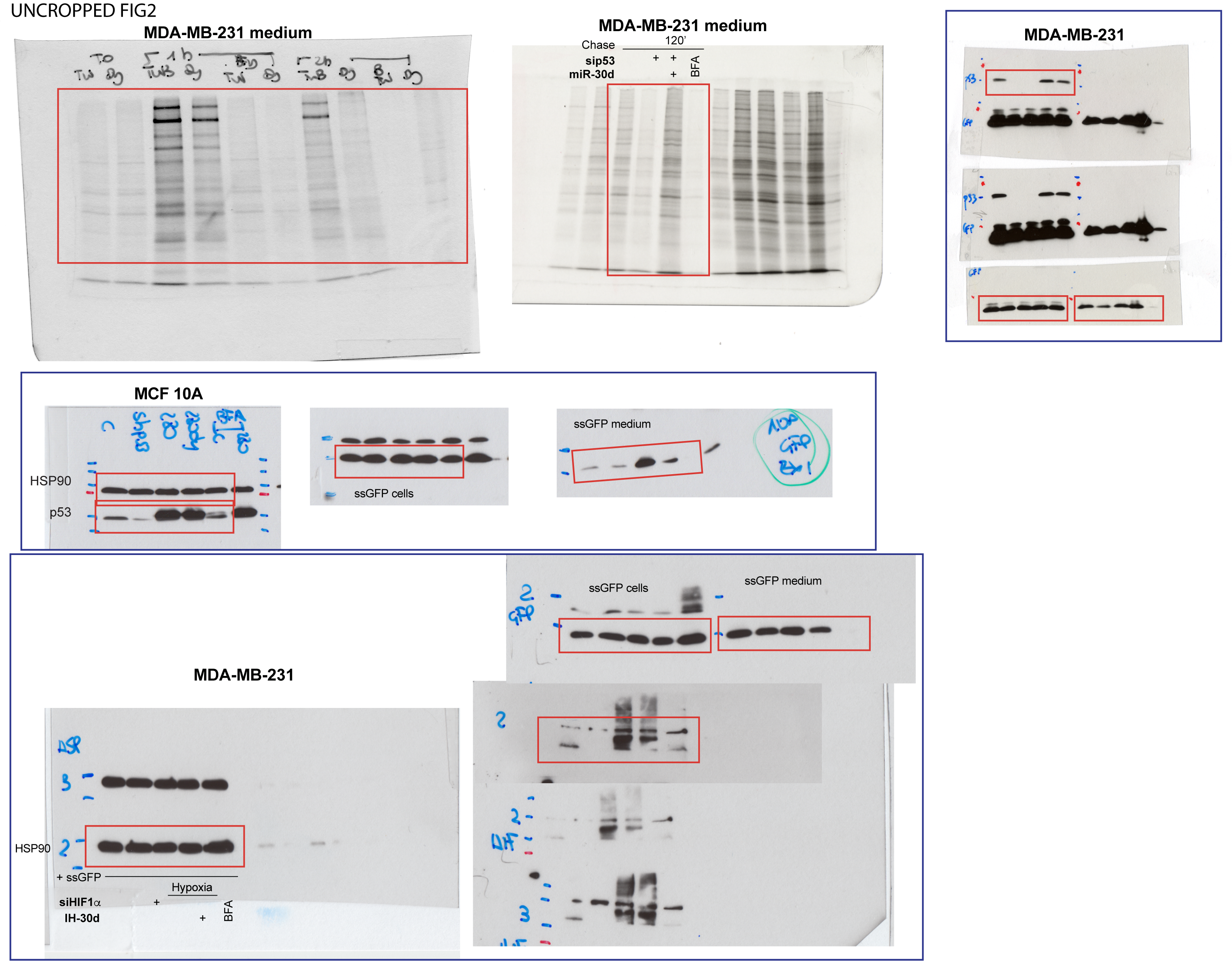

Supplement: Supplementary file 9 — Source Data File [file 41467_2020_17596_MOESM9_ESM.zip › cartella senza titolo/scan fig2.png]

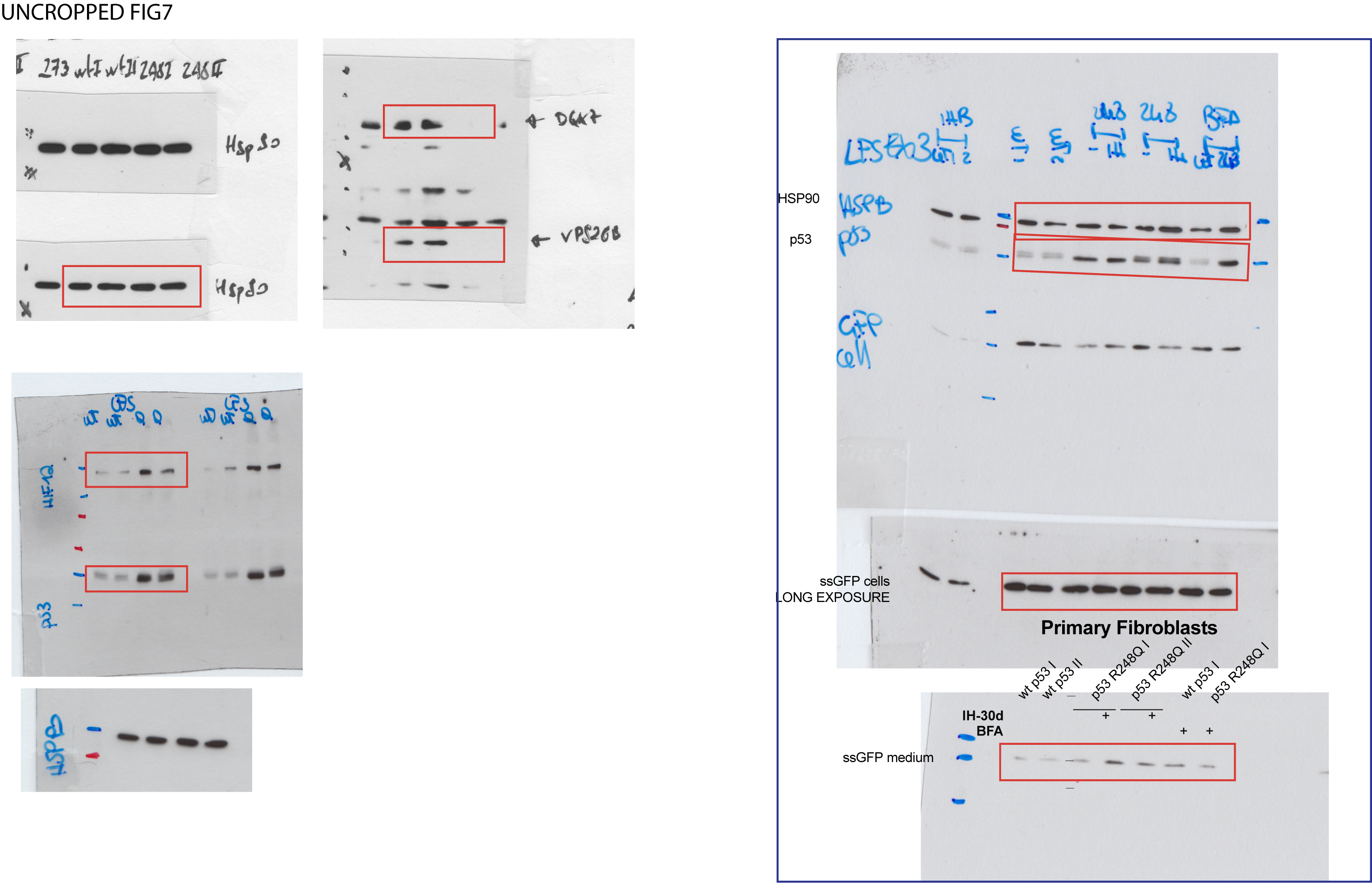

Supplement: Supplementary file 9 — Source Data File [file 41467_2020_17596_MOESM9_ESM.zip › cartella senza titolo/scan fig7.png]

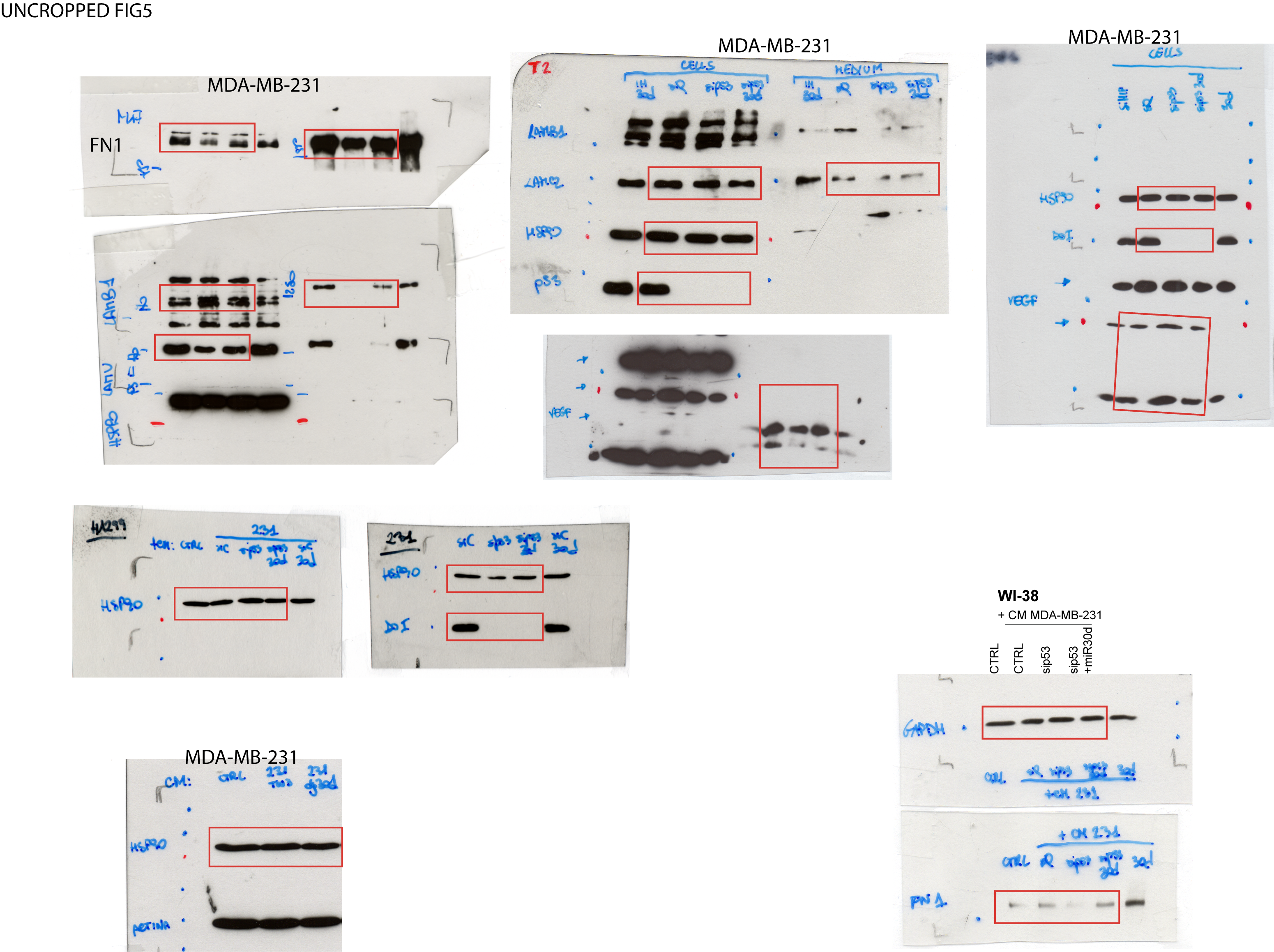

Supplement: Supplementary file 9 — Source Data File [file 41467_2020_17596_MOESM9_ESM.zip › cartella senza titolo/scan fig5.png]

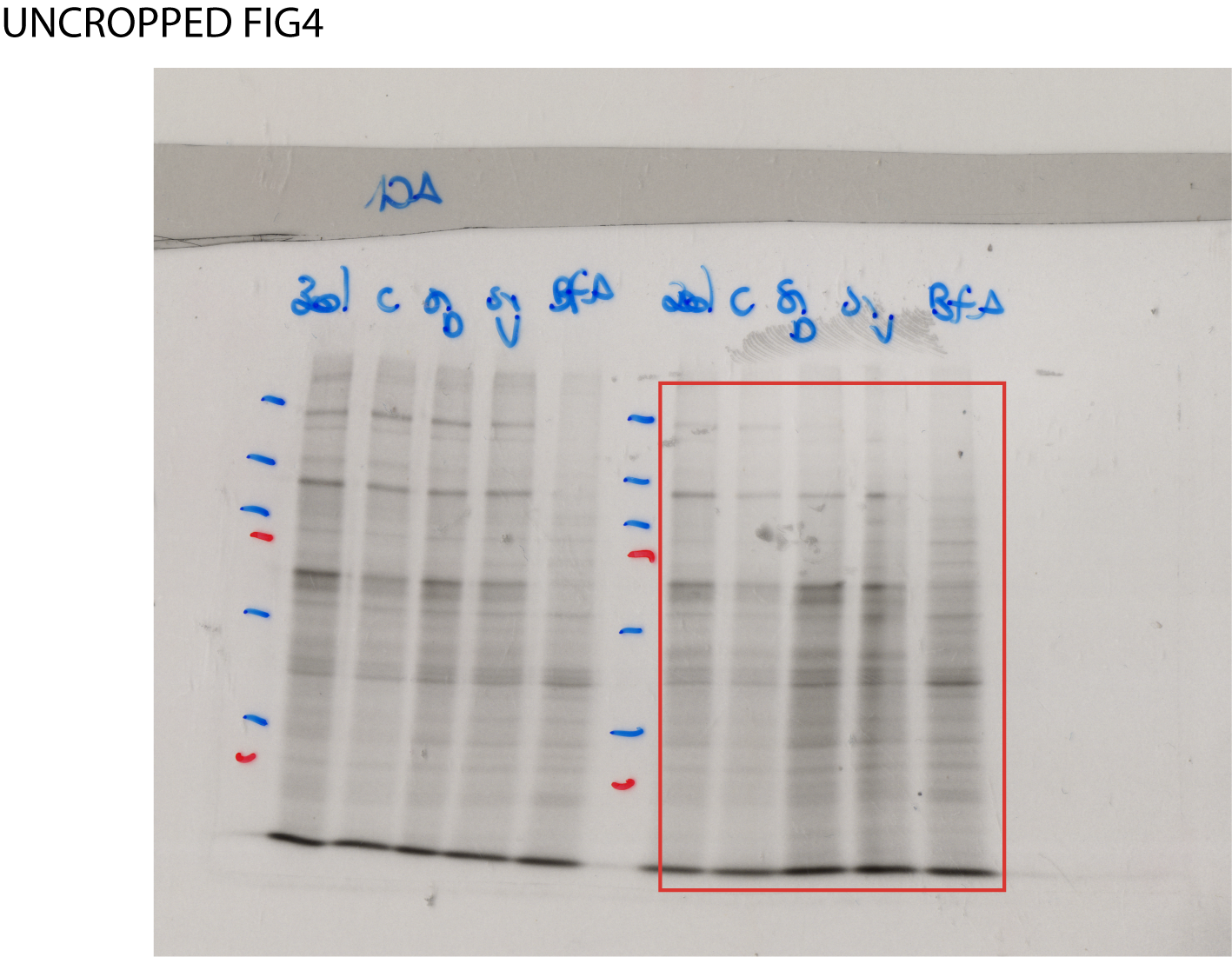

Supplement: Supplementary file 9 — Source Data File [file 41467_2020_17596_MOESM9_ESM.zip › cartella senza titolo/scan fig4.png]

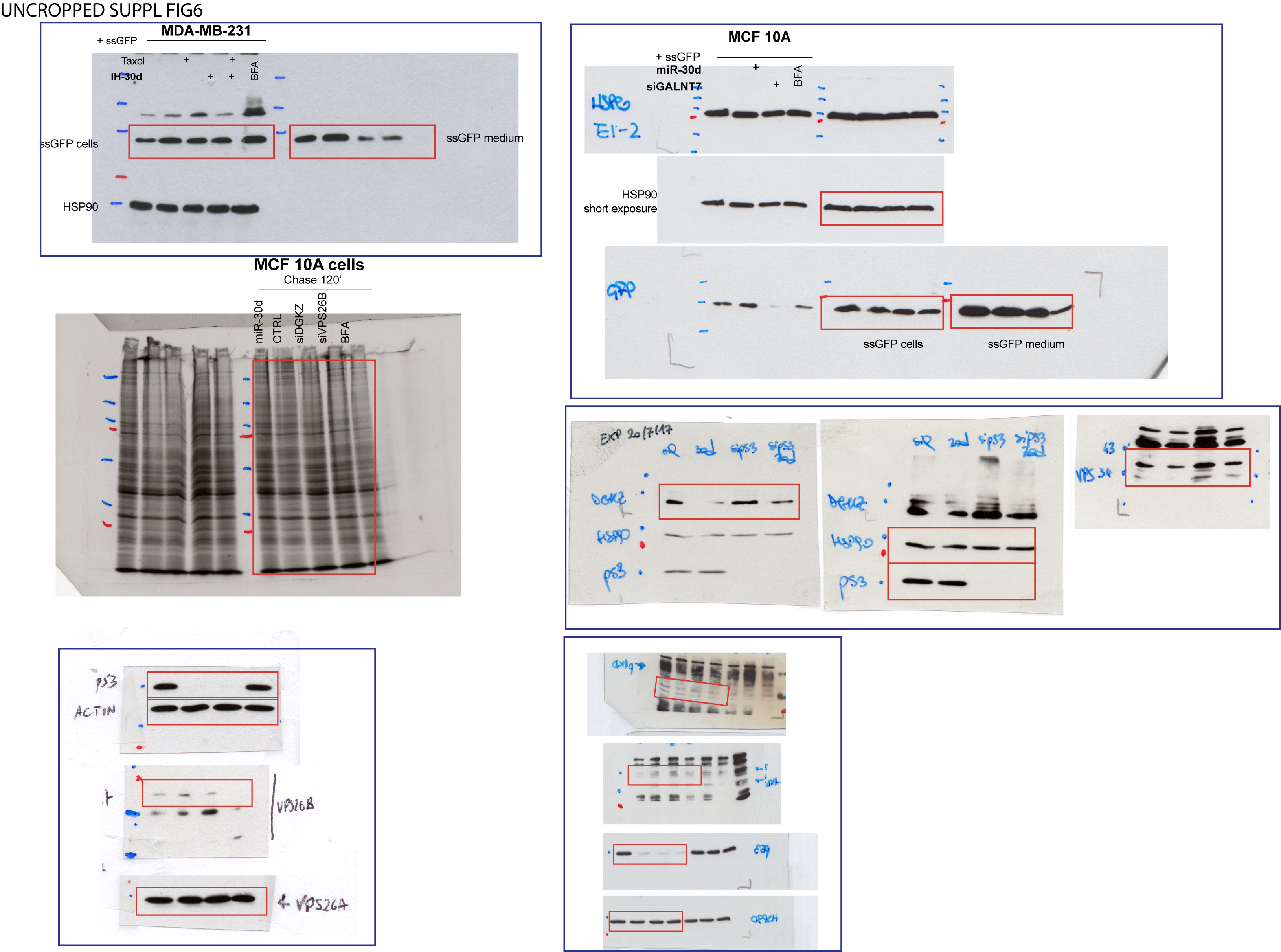

Supplement: Supplementary file 9 — Source Data File [file 41467_2020_17596_MOESM9_ESM.zip › cartella senza titolo/scan suppl fig 6.png]

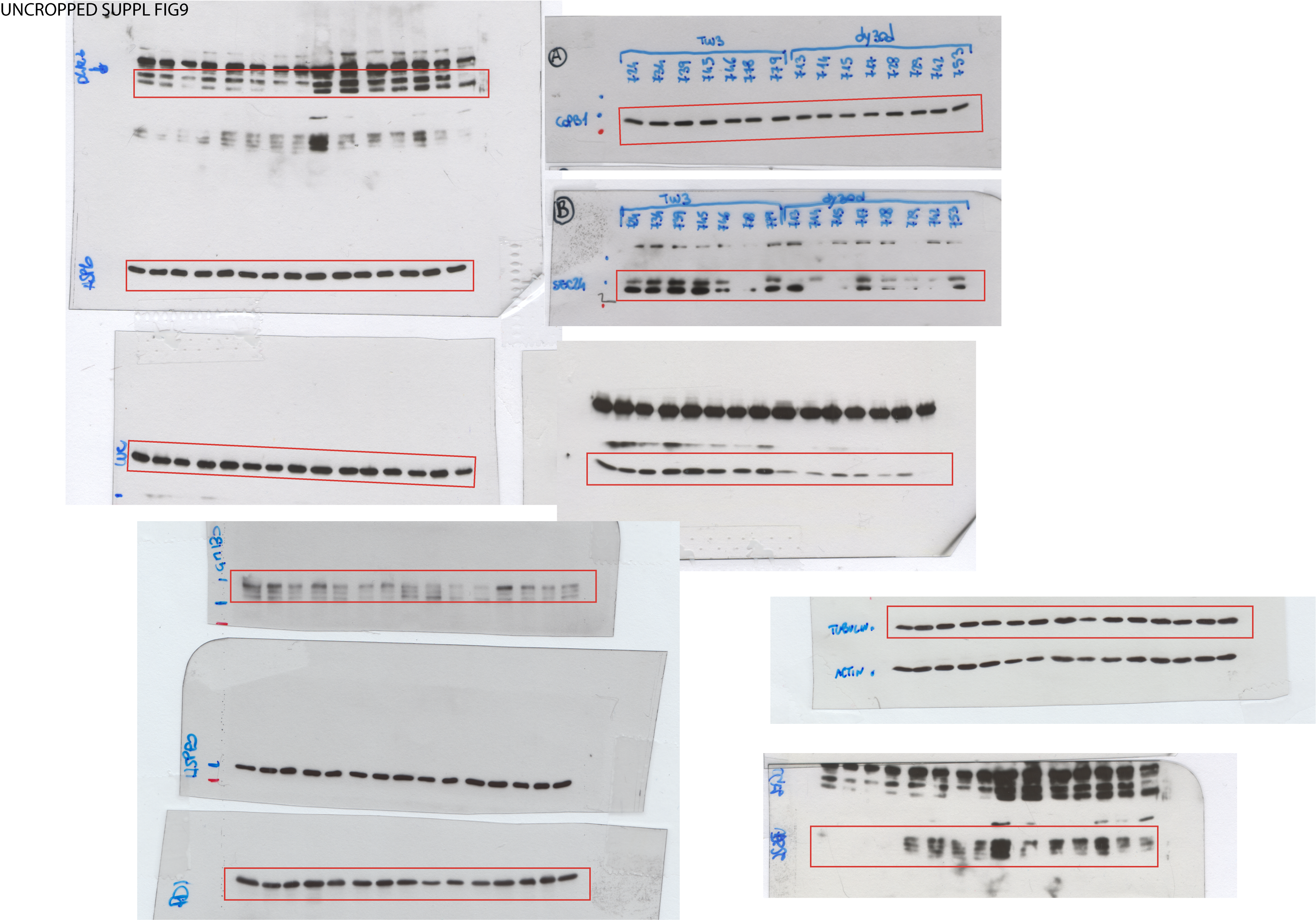

Supplement: Supplementary file 9 — Source Data File [file 41467_2020_17596_MOESM9_ESM.zip › cartella senza titolo/scan suppl fig9 .png]

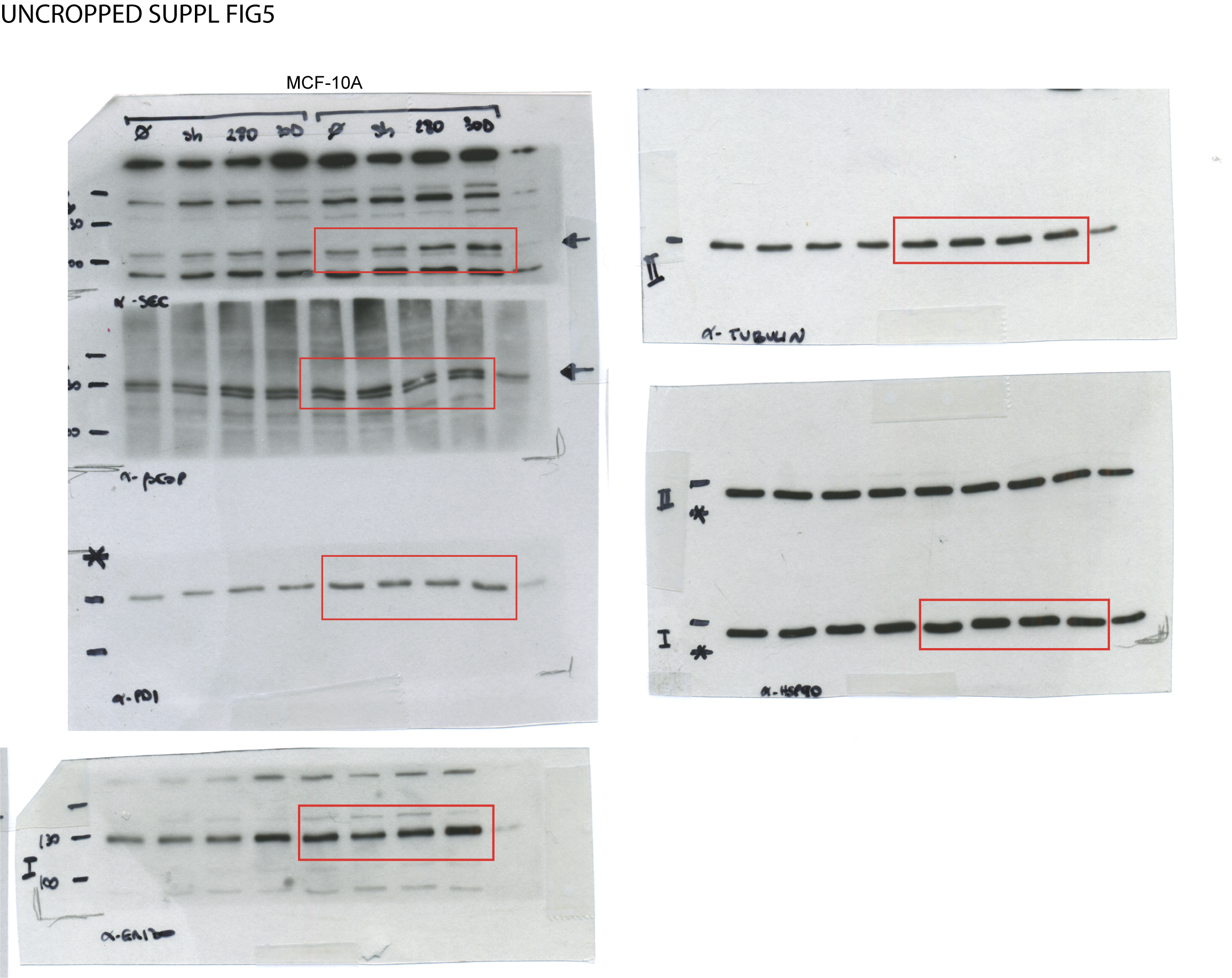

Supplement: Supplementary file 9 — Source Data File [file 41467_2020_17596_MOESM9_ESM.zip › cartella senza titolo/scan suppl fig 5.png]

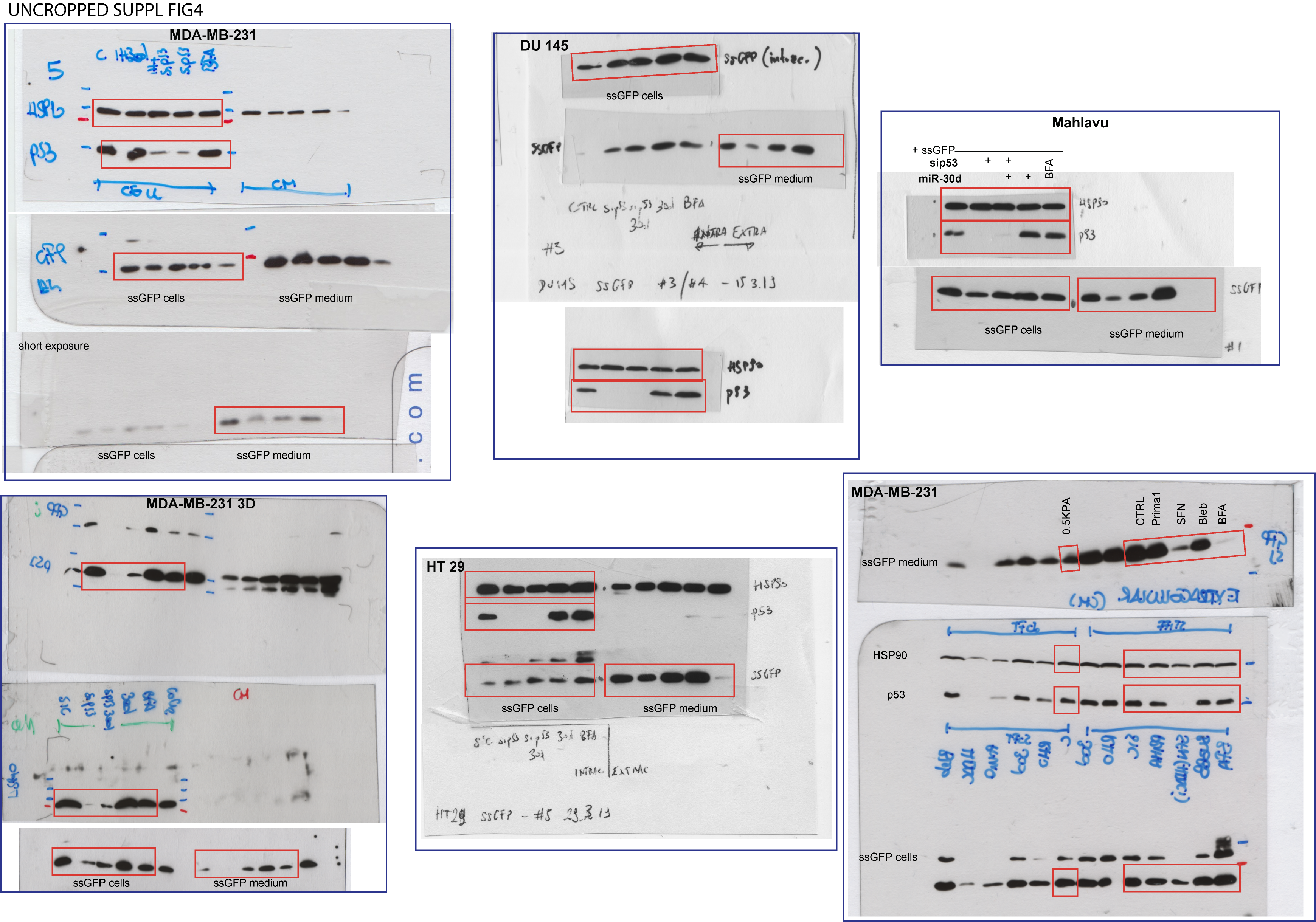

Supplement: Supplementary file 9 — Source Data File [file 41467_2020_17596_MOESM9_ESM.zip › cartella senza titolo/scan suppl fig 4.png]

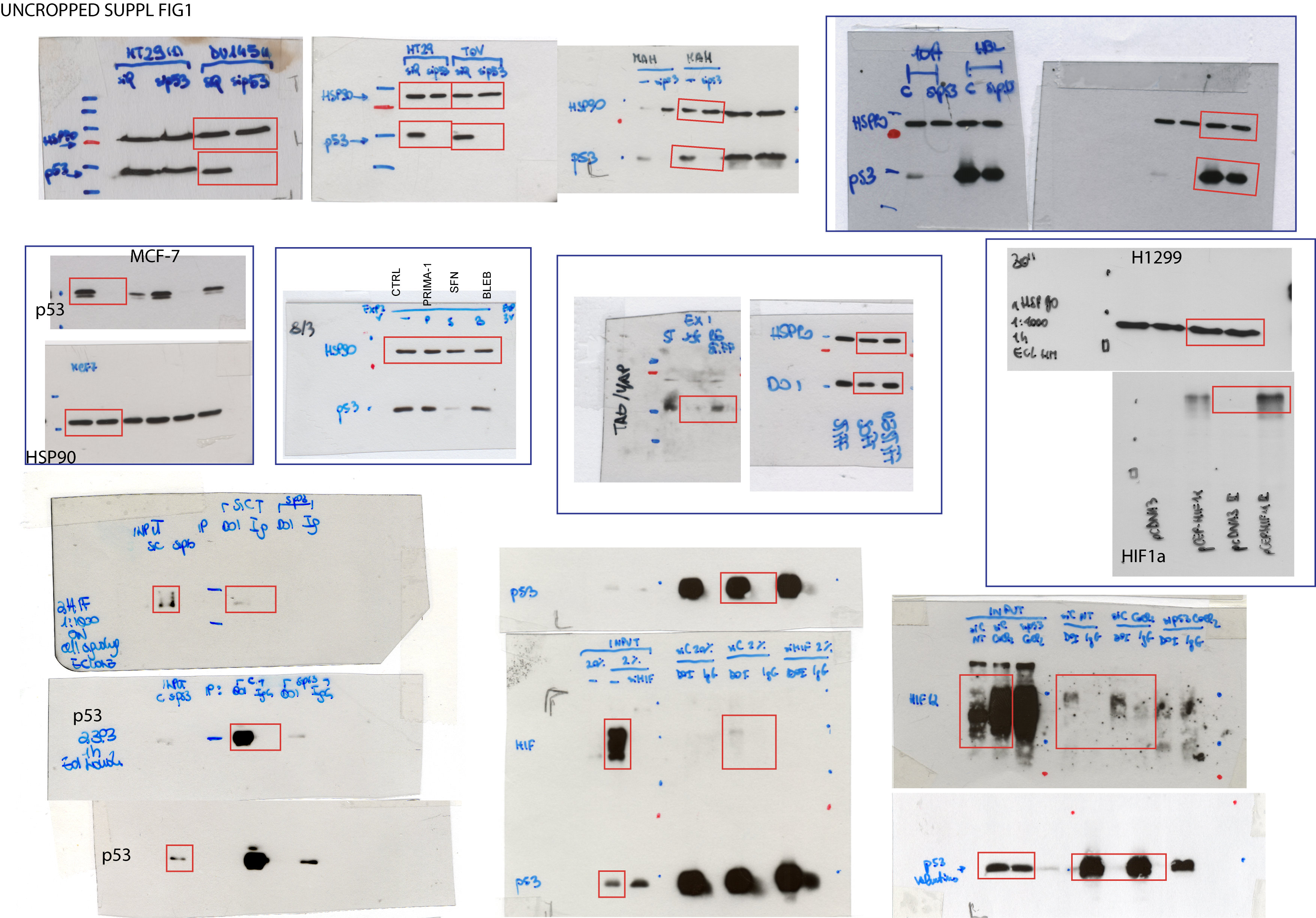

Supplement: Supplementary file 9 — Source Data File [file 41467_2020_17596_MOESM9_ESM.zip › cartella senza titolo/scan suppl fig 1.png]

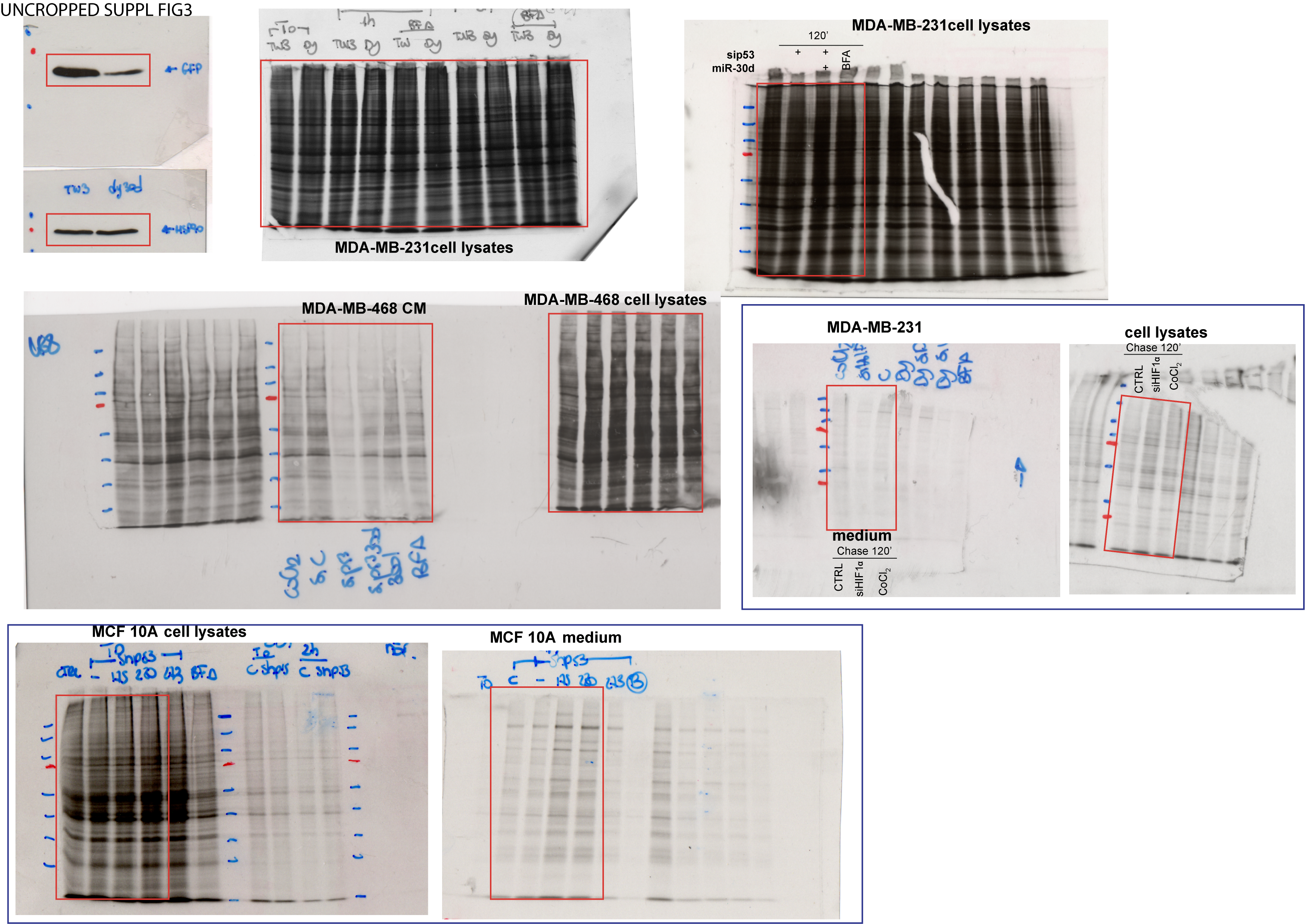

Supplement: Supplementary file 9 — Source Data File [file 41467_2020_17596_MOESM9_ESM.zip › cartella senza titolo/scan suppl fig 3.png]

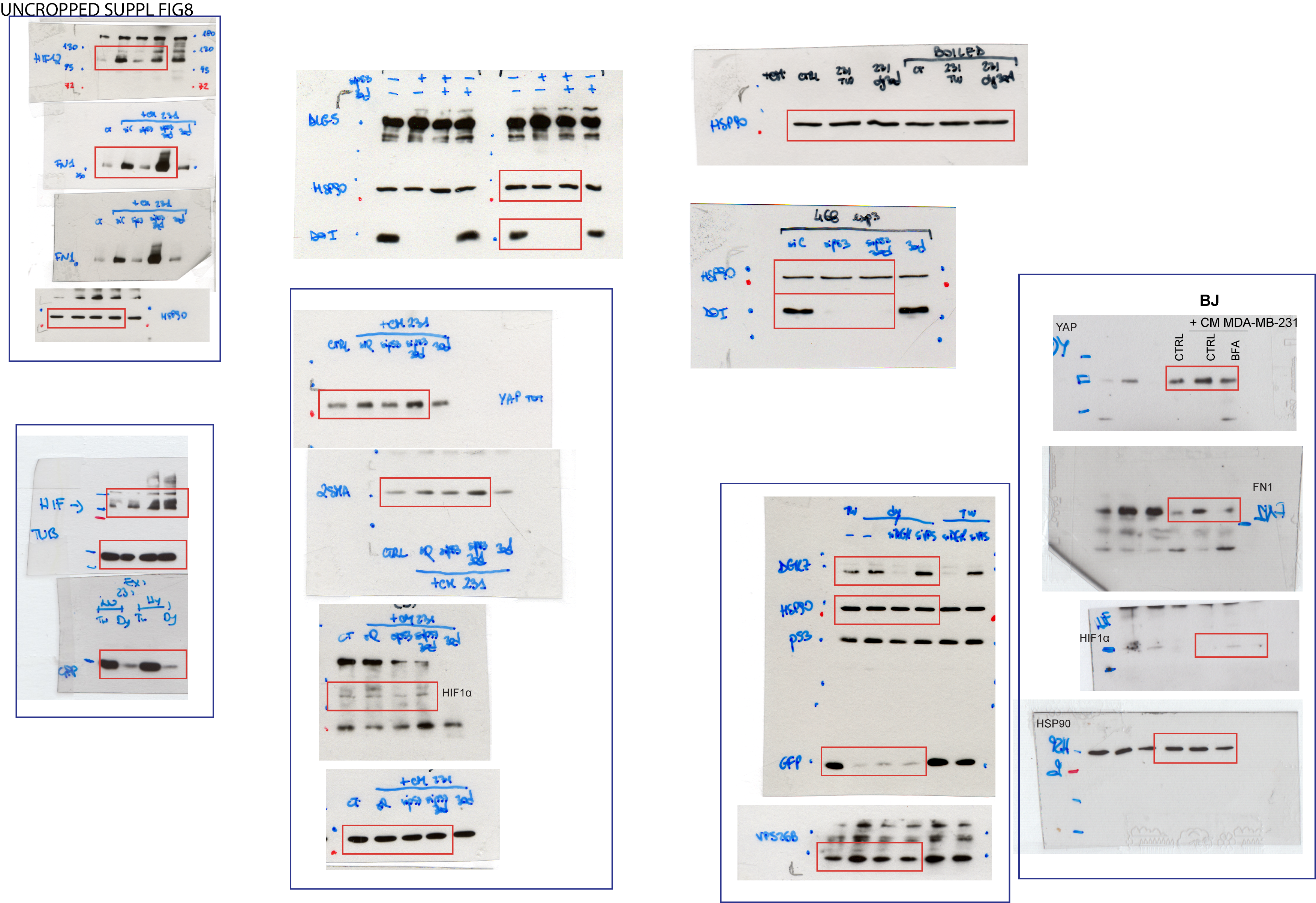

Supplement: Supplementary file 9 — Source Data File [file 41467_2020_17596_MOESM9_ESM.zip › cartella senza titolo/scan suppl fig 8ii.png]
